# Reprogramming Lesional Macrophage Homeostasis via Interferon Regulatory Factor 5 Targeted siRNA Nanoimmunotherapy for Atherosclerosis

**DOI:** 10.1021/acsnano.5c18044

**Published:** 2026-03-04

**Authors:** Zhongshan He, Yaoyao Luo, Shuping Yang, Haixing Shi, Ya-Chih Huang, Zhuoming Zhou, Shengbin Liu, Wanqin Zeng, Wei-Chieh Liu, Yongjiang Li, Yuting Chen, Duotian Qin, Xing Duan, Xi He, Wei Chen, Xiangrong Song

**Affiliations:** † Department of Clinical Pharmacy, Frontiers Science Center for Disease-related Molecular Network, State Key Laboratory of Biotherapy and Cancer Center, 12530 West China Hospital, Sichuan University, Tianfu Jincheng Laboratory, Chengdu 610041, China; ‡ Departments of Molecular Physiology and Biological Physics & Biomedical Engineering, Cardiovascular Research Center, Virginia University, Charlottesville, Virginia 22903, United States; § Laboratory of Cardiac Structure and Function, Institute of Cardiovascular Diseases, West China Hospital, Sichuan University, Chengdu 610041, China; ∥ 38017 Genomics Research Center, Academia Sinica, Taipei 115, Taiwan; ⊥ Center for Nanomedicine and Department of Anesthesiology, Brigham and Women’s Hospital, 1811 Harvard Medical School, Boston, Massachusetts 02115, United States

**Keywords:** atherosclerosis, macrophage homeostasis, small interfering RNA, nanoimmunotherapy, targeted delivery

## Abstract

Atherosclerotic macrophages predominantly exhibit a pro-inflammatory phenotype, driving chronic inflammatory and accelerating atherosclerotic progression. Interferon regulatory factor 5 (IRF5) is highly expressed in lesional macrophages within advanced atherosclerotic plaques, where it promotes the secretion of pro-inflammatory cytokines. However, current approaches lack an effective therapeutic strategy to specifically silence this gene in lesional macrophages for atherosclerosis treatment. This study aims to develop and evaluate a dual-targeted, siRNA-based nanotherapeutic platform that selectively acts on atherosclerosis-promoting genes in plaque macrophages, offering a potential strategy for treating atherosclerosis by reprogramming lesional macrophages. Here we designed and developed dual-targeted liposome-based nano-immunotherapeutics encapsulating small interfering RNA (siRNA) against IRF5 (siIRF5) to reprogram macrophage phenotypes within advanced plaques. In high-fat diet-fed *ApoE* ^–/–^ mice with advanced atherosclerotic plaques, dual-targeted siIRF5-loaded liposomes effectively accumulate within lesional macrophages, downregulate IRF5 expression, and promote anti-inflammatory macrophage polarization. Moreover, this siIRF5-based nanoimmunotherapy significantly reduces plaque burden and enhances plaque stability in two independent murine models of atherosclerosis. Furthermore, this siIRF5 nanoimmunotherapy exhibits biocompatibility even after long-term administration, underscoring its translational potential for clinical application in atherosclerosis treatment. This study introduces an innovative dual-targeted siRNA-based nanotherapeutic strategy that acts on atherosclerosis-promoting genes in plaque macrophages, offering a promising therapeutic avenue for atherosclerosis and other macrophage-driven inflammatory diseases.

## Graphical abstract

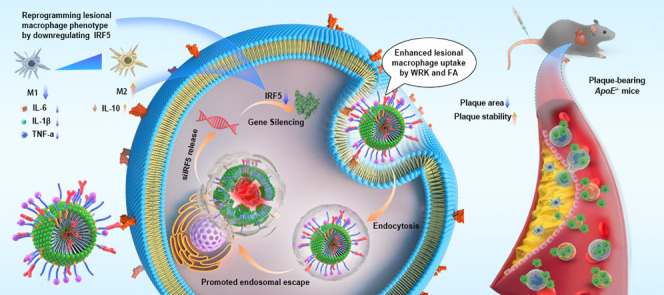

## Introduction

Atherosclerosis represents a chronic inflammatory condition in which immune cells, particularly macrophages, accumulate within the arterial intima.^1,2^ Macrophages are one of the most abundant immune cell types in atherosclerotic plaques and play crucial roles in driving the development and progression of atherosclerosis.^3,4^ Specifically, “M1-like” macrophages produce high levels of inflammatory cytokines, contributing to plaque progression and instability.^5,6^ Conversely, “M2-like” macrophages exhibit anti-inflammatory and inflammation resolution properties that are essential for plaque stabilization and regression.^7–9^ Notably, in advanced atherosclerotic plaques in both humans and mice, lesional macrophages predominantly exhibit an M1-like phenotype, characterized by chronic inflammation, enhanced plaque formation, and increased risk of rupture.^10^ This phenotypic dominance contributes to necrotic core expansion and heightens the likelihood of atherothrombotic events such as myocardial infarction and stroke.^11^ Therefore, reprogramming lesional macrophages from a pro-inflammatory (M1-like) to an anti-inflammatory (M2-like) phenotype is considered a promising approach for atherosclerosis.^12,13^ Indeed, various attempts have been made to modulate macrophage phenotypes through systemic treatment with anti-inflammatory agents, such as methotrexate and monoclonal antibodies targeting interleukin-1β (IL-1β),^14,15^ as demonstrated in the CANTOS trial (NCT01327846) and the Cardiovascular Inflammation Reduction Trial (CIRT; NCT01594333). However, these therapeutic strategies are often associated with suboptimal efficacy, and undesirable systemic adverse effects, including excessive immunosuppression and an increased risk of infection.^1,6^ These limitations underscore the urgent need for precision therapeutic strategies that can selectively modulate macrophage phenotypes within atherosclerotic lesions while minimizing systemic toxicity.

Aberrant activation of inflammatory signaling cascades in plaque-resident macrophages sustains local inflammatory amplification and drives atherosclerotic progression. Among these pathways, the transcription factor interferon regulatory factor 5 (IRF5) is markedly upregulated in lesional macrophages within advanced atherosclerotic plaques in both human patients and mouse models.^16,17^ IRF5 promotes atherogenesis by driving M1-like polarization and enhancing the secretion of pro-inflammatory cytokines in these macrophages.^18–22^ Despite its critical role, current therapeutic strategies lack the ability to effectively and selectively target gene expression in lesional macrophages, representing a major unmet need in the treatment of atherosclerosis.

RNA interference (RNAi) harnesses small interfering RNAs (siRNAs) to achieve precise post-transcriptional gene silencing with minimal off-target effect, highlighting RNAi as a compelling therapeutic strategy for cardiovascular diseases.^23–25^ Notably, the siRNA-based drug (Leqvio; inclisiran), which targets PCSK9, has received U.S. Food and Drug Administration approval for the treatment of atherosclerotic cardiovascular disease, underscoring the clinical potential of RNAi therapeutics.^24^ While this liver-targeted strategy effectively reduces systemic low-density-lipoprotein cholesterol (LDL-C), directly modulating lesional macrophage phenotypes to resolve inflammation and limit plaque progression remains an unmet medical need. In this context, designing sequence-specific siRNAs to suppress IRF5 expression in lesional macrophages represents an approach to reprogram pro-inflammatory macrophage toward anti-inflammatory phenotype. Such immunogene therapy holds significant potential for achieving targeted resolution of vascular inflammation and promoting plaque stabilization. However, despite their promise, siRNA-based nanomedicines face several challenges that hinder their clinical translation, including rapid degradation, short circulation time, poor cellular uptake, limited accumulation in atherosclerotic plaques, and inefficient endosomal escape.^26–28^

To address these barriers, we propose the development of engineered IRF5 siRNA (siIRF5) nano-immunotherapeutics to reprogram macrophage in advanced plaques for atherosclerosis therapy. The engineered nanoplatform is based on PEGylated cationic liposomes encapsulating siIRF5; the liposomal structure effectively protects siRNA from enzymatic degradation by shielding it from serum nucleases, while PEGylation prolongs circulation by evading recognition and clearance by the mononuclear phagocyte system (MPS).^26–30^ To further enhance delivery efficiency and specificity toward lesional macrophages in atherosclerotic plaques, we functionalized the liposome surface with WRK—a cyclic cell-penetrating peptide^31^—and folic acid (FA)—a cell membrane-targeting molecule. WRK facilitates deep tissue penetration and promotes endosomal escape, thereby improving cytoplasmic siRNA availability.^31–35^ Simultaneously, FA targets folate receptor-β, which is overexpressed on lesional macrophages, enabling selective cellular uptake and enhanced macrophage targeting.^36,37^ These dual-ligand modifications significantly improve the precision and effectiveness of siRNA delivery to atherosclerotic macrophages. We demonstrate that dual-ligand-functionalized siIRF5 nano-immunotherapeutics (FW-LP@siIRF5, Figure 1) efficiently accumulate in lesional macrophages of *ApoE* ^–/–^ mice with advanced atherosclerosis. Moreover, our findings show that FW-LP@siIRF5 treatment effectively silences IRF5 expression, promotes anti-inflammatory macrophage polarization, and significantly reduces plaque size while enhancing plaque stability in two independent murine models of atherosclerosis. Furthermore, this dual-ligand-functionalized siIRF5 nanoimmunotherapy exhibits biocompatibility even after long-term administration. Our findings establish a nanotherapeutic strategy for immune reprogramming in atherosclerotic and offers a broadly applicable platform for treating other macrophage-driven inflammatory diseases.

**Figure 1:**
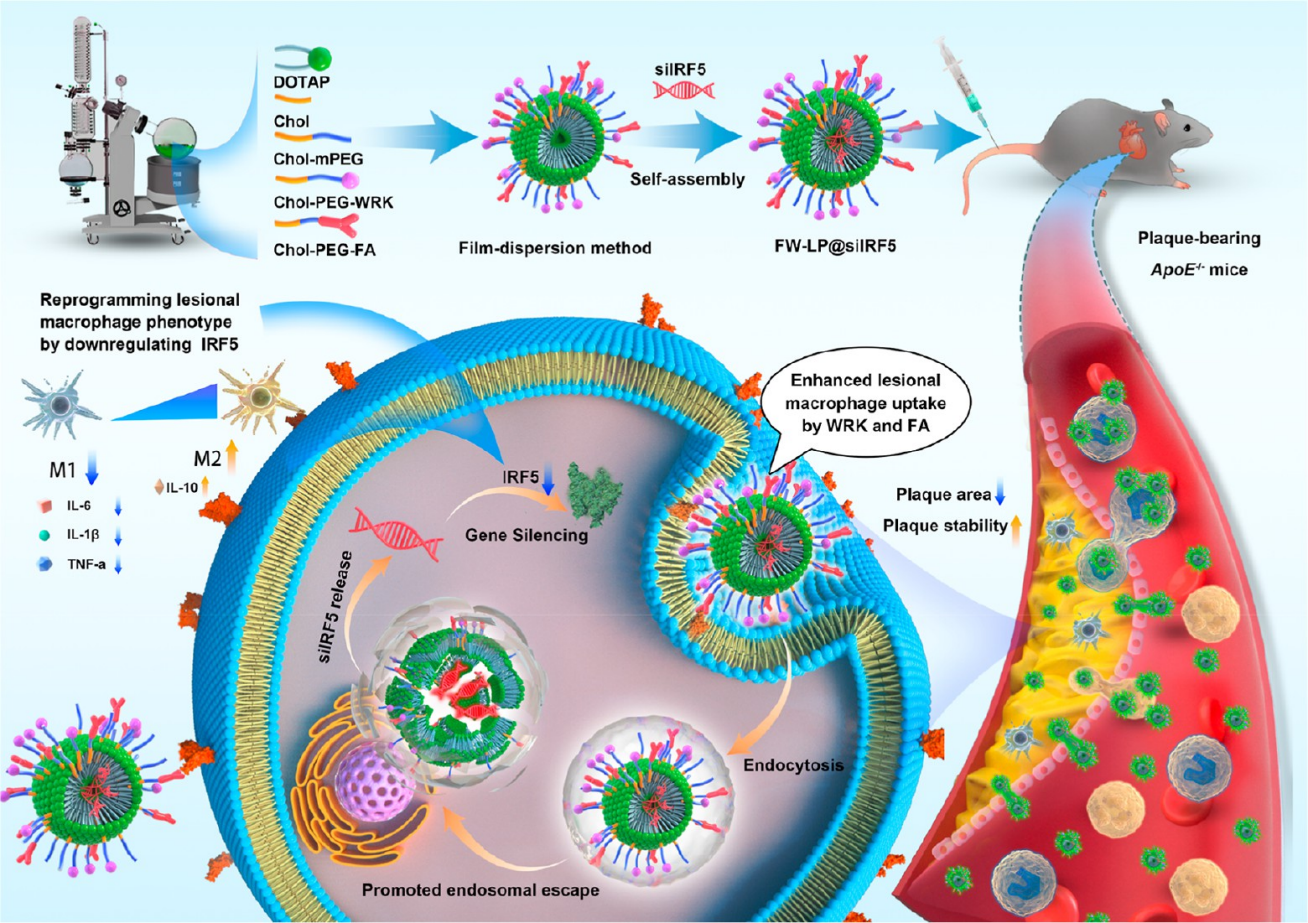
Schematic illustration of the synthetic strategy and antiatherosclerotic mechanism of FW-LP@siIRF5 FW-LP@siIRF5 was synthesized using a robust thin-film dispersion method and engineered as a dual-ligand-functionalized nanoplatform for targeted siRNA delivery to lesional macrophages in atherosclerotic plaques. Following systemic administration, FW-LP@siIRF5 efficiently accumulates in plaque macrophages in *ApoE* ^–/–^ mice, where it silences IRF5 expression, reprograms pro-inflammatory macrophages (M1-like) into an anti-inflammatory (M2-like) phenotype, thereby promoting resolution of inflammation. The resulting immunomodulation leads to reduced plaque burden and improved plaque stability. Notably, FW-LP@siIRF5 also exhibits biocompatibility during prolonged administration. This study presents a promising siRNA-based immunotherapeutic approach for atherosclerosis and highlights the broader potential of the targeted nucleic acid delivery system for treating other macrophage-driven inflammatory diseases.

## Results

### Study Summary

Figure 1 shows a schematic illustration of the synthetic strategy and antiatherosclerotic mechanism of FW-LP@siIRF5. We began with the design and characterization of FW-LP@siIRF5, and the cellular uptake, lysosomal escape, and gene silencing efficiency of FW-LP@siIRF5 in macrophages. Next, we evaluated the repolarization of macrophage phenotypes and the anti-inflammatory ability of FW-LP@siIRF5 in vitro. Subsequently, we assessed the pharmacokinetics, biodistribution, atherosclerotic plaque-targeting capability, and antiatherosclerotic efficacy of FW-LP@siIRF5 in an *ApoE* ^–/–^ mouse model of atherosclerosis. Finally, the therapeutic mechanisms and safety profiles of FW-LP@siIRF5 were investigated.

### Synthesis and Characterization of FW-LP@siIRF5

We utilized a cationic liposome-mediated delivery platform to encapsulate siRNA through a robust thin-film dispersion method (Figure 1).^38^ To enhance both cell membrane-penetrating and macrophage-targeting efficiency, we functionalized the surface of the liposomal nanoplatform with two synthetic ligands: WRK-PEG_2k_-Chol (WRK, Figure S1) and FA-PEG_2k_-Chol (FA, Figure S2). These ligands were anchored onto the phospholipid-like bilayer via cholesterol moieties, enabling stable incorporation into the liposome membrane (Figure 1). The resulting functionalized liposomes were designated as FW-LP. As a control, we synthesized PEG-Chol-coated cationic liposomes (PEG-LP), which lacks the FA and WRK ligands, to evaluate the cell membrane-penetrating and macrophage-targeting performance of FW-LP in comparison.

To generate therapeutic nanocarriers, IRF5 siRNA (siIRF5) was subsequently encapsulated into both FW-LP and PEG-LP via electrostatic self-assembly, forming FW-LP@siIRF5 (Figure 2A) and PEG-LP@siIRF5, respectively. Dynamic light scattering (DLS) and transmission electron microscopy (TEM) were employed to characterize the physicochemical properties of the siIRF5-loaded liposomes (Figure 2B–D and Table S1). As shown in Figure 2B and Table S1, the average hydrodynamic diameters of FW-LP@siIRF5 and PEG-LP@siIRF5 were 120.4 ± 10.8 nm and 102.7 ± 6.2 nm, respectively—slightly larger than their unloaded counterparts—indicating successful siRNA loading. Zeta potential measurements further confirmed successful siRNA encapsulation. The zeta potentials of FW-LP@siIRF5 and PEG-LP@siIRF5 were 5.4 ± 1.4 mV and 4.2 ± 1.1 mV, respectively, both significantly lower than those of the unloaded FW-LP (15.1 ± 5.4 mV) and PEG-LP (13.2 ± 4.1 mV) (Figure 2C and Table S1). Moreover, the modest increase in size observed in FW-LP@siIRF5 compared to PEG-LP@siIRF5 was attributed to the additional surface coating with WRK-PEG2k-Chol and FA-PEG2k-Chol (Table S1). Morphological analysis by TEM revealed that FW-LP@siIRF5 exhibited a uniform, free-standing spherical shape (Figure 2D). Time-course DLS measurements (Figure S3A) demonstrated that FW-LP@siIRF5 maintained colloidal stability under physiological conditions.

**Figure 2:**
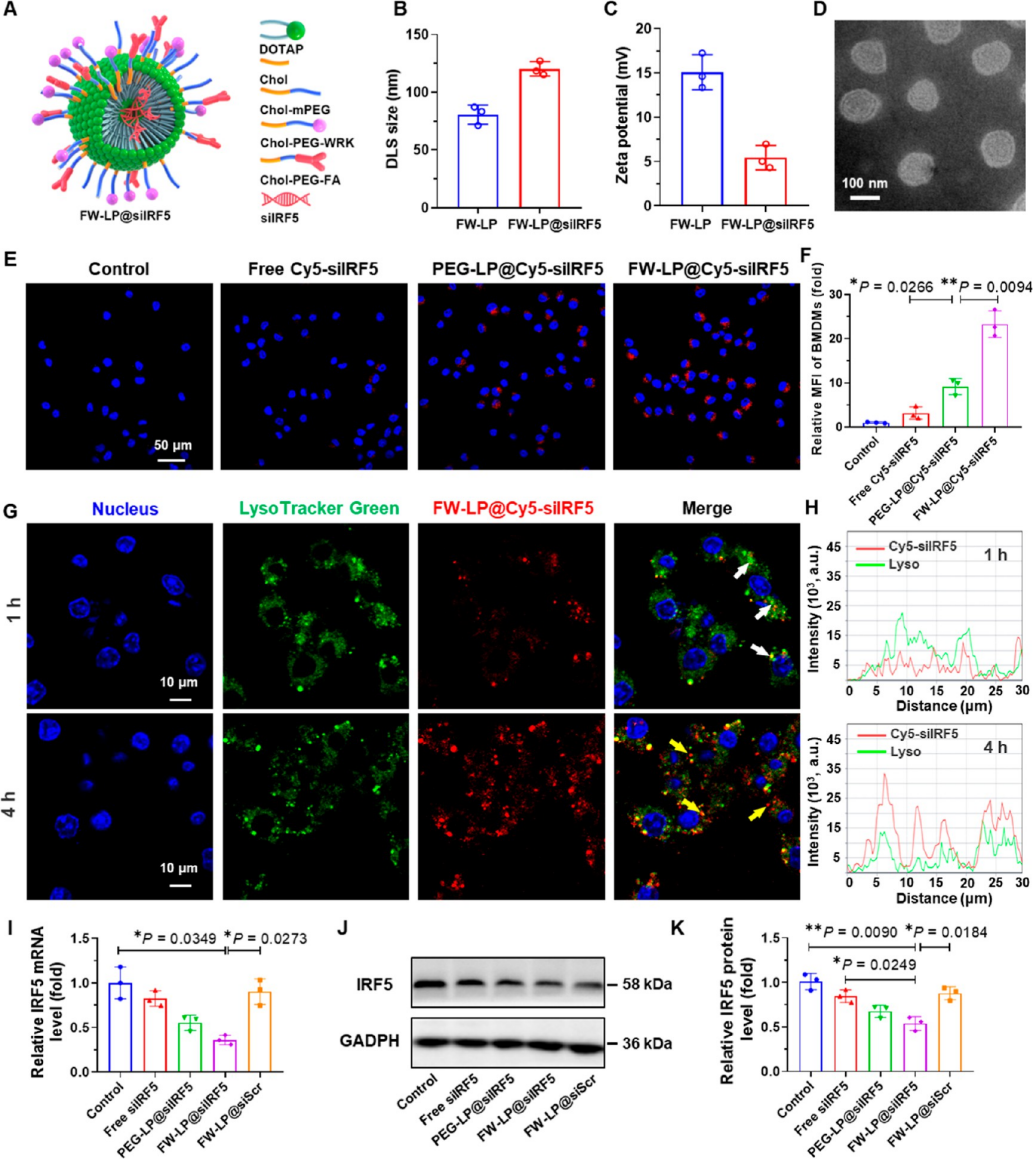
Characterization and in vitro studies of FW-LP@siIRF5. (A) Schematic illustration of the dual-ligand-functionalized FW-LP@siIRF5 design. (B,C) Hydrodynamic diameter and zeta potential, as determined by dynamic light scattering (DLS), of empty FW-LPs (without siIRF5) and FW-LP@siIRF5 (*n* = 3 independent samples, mean ± S.D.). (D) Transmission electron microscopy image of FW-LP@siIRF5 negatively stained with uranyl acetate (scale bar, 100 nm). (E) Confocal microscopy images showing cellular uptake of Cy5-labeled siIRF5 delivered via free siRNA, PEG-LP@siIRF5, and FW-LP@siIRF5 into bone marrow-derived macrophages (BMDMs) after 4 h incubation. Nuclei stained with DAPI (blue). Scale bars, 50 μm. (F) Quantification of BMDM uptake by flow cytometry, shown as mean fluorescence intensity (MFI) ratio relative to control (*n* = 3 independent samples, mean ± S.D.). (G) Confocal microscopy images showing lysosomal escape of FW-LP@siIRF5 in BMDMs after 1 or 4 h incubation. Cy5-siIRF5 (red), nuclei (DAPI, blue), and lysosomes (LysoTracker Green, green). Scale bars, 10 μm. (H) Quantitative analysis of fluorescence intensities inside (white arrows) and outside (yellow arrow) lysosomes from panel G. (I–K) In vitro gene silencing efficacy of FW-LP@siIRF5 and controls in LPS/INF-γ-stimulated BMDMs, assessed 48 h post transfection. (I) Relative IRF5 mRNA expression level measured by RT-qPCR (*n* = 3 independent samples, mean ± S.D.). (J,K) IRF5 protein levels evaluated and quantified by Western blotting on LPS/INF-γ-stimulated BMDMs pretreated with various siRNA formulations (*n* = 3 independent samples, mean ± S.D.). FW-LPs loaded with scrambled siRNA (FW-LP@siScr) served as a control. Additionally, PEG-LP@siIRF5 was included to assess the additive effects of WRK (cell-penetrating peptide) and FA (targeting ligand). Statistical significance was determined using one-way ANOVA with a Games-Howell post hoc test. *P < 0.05, and **P < 0.01.

To evaluate encapsulation efficiency, Cy5-labeled siIRF5 was used. The encapsulation efficiencies were 89.0 ± 6.3% for FW-LP and 82.3 ± 6.5% for PEG-LP, with no significant difference (Figure S3B,C), indicating efficient siIRF5 loading and encapsulation stability in both FW-LP and PEG-LP formulations. This high loading capacity is likely driven by strong electrostatic interactions between the cationic lipid DOTAP and the anionic phosphate backbone of siRNA, as well as physical confinement within the liposomal core.

To further assess protection of siRNA against nuclease degradation, a serum stability assay was conducted. FW-LP@siIRF5 nanoparticles were incubated with serum at 37 °C for 4, 8, 12, and 24 h, followed by electrophoretic mobility shift assays. While free siRNA was rapidly degraded, siRNA extracted from FW-LP@siIRF5 remained intact even after 24 h (Figure S4), indicating robust protection by the liposomal nanocarriers. In addition, we evaluated the release kinetics of FW-LP@siIRF5 (Figure S5) under standard physiological conditions (pH 7.4, H_2_O_2_ = 0 mM) and plaque-mimicking conditions (pH 6.3, H_2_O_2_ = 1.0 mM).^6^ The release profiles showed substantially higher siIRF5 release under plaque-mimicking conditions. This accelerated release is likely due to mild acidity and elevated ROS, which can destabilize lipid membranes and promote siRNA diffusion.

Collectively, FW-LP@siIRF5 demonstrates siRNA loading efficiency, high colloidal stability, and effective protection against nuclease-mediated degradation, underscoring its potential as a promising nanocarrier for gene silencing in atherosclerosis therapy.

### Cellular Uptake, Lysosomal Escape and Gene Silencing Efficiency of FW-LP@siIRF5 In Vitro

Efficient siRNA-mediated gene silencing requires two key prerequisites: high cellular uptake and successful endosomal escape, allowing siRNA to reach the cytoplasmic RNA-induced silencing complex (RISC).^39–41^ To evaluate these criteria, Cy5-labeled IRF5 siRNA (Cy5-siRNA) was encapsulated into liposomal formulations and their cellular uptake in bone marrow-derived macrophages (BMDMs) was assessed by confocal microscopy images (Figure 2E) and flow cytometry analysis (Figure 2F). Both FW-LP@Cy5-siIRF5 and PEG-LP@Cy5-siIRF5 were efficiently internalized by BMDMs, whereas free Cy5-siIRF5 exhibited negligible uptake (Figure 2E,F). Notably, FW-LP@Cy5-siIRF5 exibited substantially higher intracellular accumulation than PEG-LP@Cy5-siIRF5, likely due to the synergistic effects of the FA-mediated macrophage targeting and WRK-facilitated cell membrane penetration. These findings suggest that the dual-ligand surface modification of FW-LP markedly enhances macrophage uptake efficiency. Furthermore, uptake pathway analyses indicate that FW-LP@siIRF5 enters macrophages predominantly via clathrin-mediated endocytosis, with limited involvement of caveolae-dependent pathways and minimal contribution from macropinocytosis (Figure S6).

To assess lysosomal escape, BMDMs were stained with LysoTracker (green) and incubated with FW-LP@Cy5-siIRF5. After 1 h of incubation, confocal microscopy revealed colocalization of the red (Cy5-siIRF5) and green (lysosomal) fluorescence signals (Figure 2G), indicating initial entrapment of the FW-LP@Cy5-siIRF5 within lysosomes. By 4 h postincubation, the red fluorescence (FW-LP@Cy5-siIRF5) was observed to have separated from the green signal, suggesting successful endosomal escape of FW-LP@Cy5-siIRF5 into the cytoplasm (Figure 2G). Additionally, we quantified the fluorescent intensities of FW-LP@Cy5-siIRF5 inside the lysosomes (white arrow, 1 h) and outside the lysosomes (yellow arrow, 4 h) (Figure 2H). The results further confirmed that the FW-LP@Cy5-siIRF5 demonstrated effective endosomal escape.

Encouraged by the high cellular uptake and efficient lysosomal escape, we next evaluated the gene silencing efficiency of FW-LP@siIRF5. BMDMs were stimulated with LPS and IFN-γ, then treated with various siRNA formulations for 48 h. Quantitative RT-PCR (Figure 2I) and Western blot analysis (Figure 2J,K) showed that FW-LP@siIRF5 achieved significant downregulation of IRF5 expression—approximately 62% reduction at the mRNA level and 47% reduction at the protein level—compared to untreated or scrambled siRNA (FW-LP@siScr) controls. Importantly, FW-LP@siScr did not significantly alter IRF5 expression, confirming the sequence-specific silencing activity of siIRF5. Moreover, FW-LP@siIRF5 exhibited no significant cytotoxicity in BMDMs, vascular smooth muscle cells (VSMCs), human umbilical vein–derived endothelial cells (HUVECs), or plaque-derived primary VSMCs and mouse aortic endothelial cells (MAEC), relevant to atherosclerosis, as demonstrated in viability assays (Figure S7), highlighting its favorable biocompatibility.

Taken together, these results demonstrate that FW-LP@siIRF5 efficiently delivers siRNA to macrophages, enables effective lysosomal escape, and achieves robust gene silencing with minimal cytotoxicity. These properties make FW-LP@siIRF5 a promising candidate for further development in atherosclerosis-targeted RNAi therapy.

### FW-LP@siIRF5 Repolarizes the Macrophage Phenotype In Vitro

Reprogramming macrophages toward an anti-inflammatory M2-like phenotype is critical for resolving inflammation and promoting plaque regression.^7,10–12^ Given this paradigm, we investigated the macrophage polarization capability of FW-LP@siIRF5 by measuring the expression of CD206^+^ (M2-like macrophage marker) and iNOS^+^ (M1-like macrophage marker) in LPS/INF-γ-stimulated BMDMs. Flow cytometry analysis (Figure 3A–C) revealed that FW-LP@siIRF5 treatment significantly increased the proportion of CD206^+^ cells in F4/80^+^ BMDMs (Figure 3A–C), indicating enhanced M2-like polarization. In contrast, the population of F4/80^+^iNOS^+^ pro-inflammatory macrophages was markedly reduced following FW-LP@siIRF5 treatment (Figure 3A,B,D), suggesting potent suppression of M1-like phenotypes. Moreover, compared with nontargeted PEG-LP@siIRF5 or scrambled siRNA-loaded FW-LP (FW-LP@siScr), FW-LP@siIRF5 exhibited superior efficacy in promoting the anti-inflammatory M2-like phenotype (F4/80^+^CD206^+^) while concurrently reducing the pro-inflammatory M1-like phenotype (F4/80^+^iNOS^+^) (Figure 3C,D). These results were further supported by confocal microscopy images (Figure S8), which also showed increased CD206 and decreased iNOS fluorescence in FW-LP@siIRF5-treated macrophages, consistent with flow cytometry findings. Moreover, quantitative real-time PCR (qRT-PCR) analyses confirmed that FW-LP@siIRF5 treatment significantly promoted macrophage repolarization toward a reparative M2 phenotype by modulating the PPAR (PPAR-γ) pathway as well as key inflammatory signaling pathways, including NF-κB and STAT1/6 (Figure S9 and Table S2). To further confirm the anti-inflammatory effects of FW-LP@siIRF5 following macrophage repolarization, we measured the secretion of key cytokines. FW-LP@siIRF5 treatment markedly suppressed the secretion of pro-inflammatory cytokines, including TNF-α, IL-1β, and IL-6, in LPS/IFN-γ-stimulated BMDMs (Figure 3E), while concomitantly enhancing the expression of the anti-inflammatory cytokine IL-10 (Figure 3F), compared with other treatment groups.

**Figure 3:**
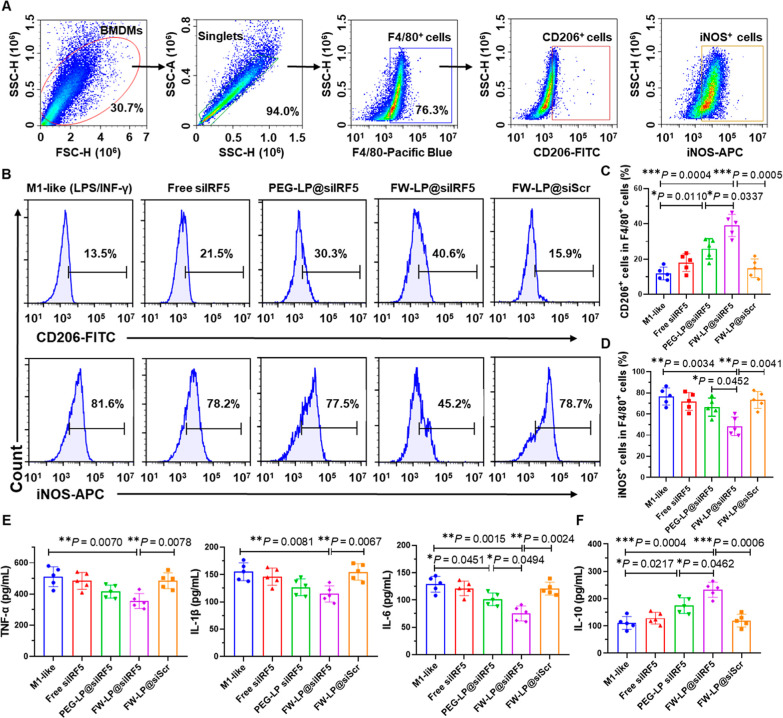
FW-LP@siIRF5 reprograms macrophage phenotype in an inflammatory microenvironment in vitro. (A–D) Flow cytometry analysis of the M2-like (CD206^+^) and M1-like (iNOS^+^) macrophage markers on F4/80^+^ BMDMs. BMDMs were pretreated with LPS (50 ng/mL) and IFN-γ (50 ng/mL) for 12 h to induce M1 polarization, followed by treatment with medium (M1 control) or various siRNA formulations for an additional 24 h. (A) Gating strategy used for the analysis of the CD206^+^ and iNOS^+^ expression within BMDMs. (B) Representative flow cytometry histograms. (C,D) Quantitative analysis of CD206^+^ (C) and iNOS^+^ (D) populations in F4/80^+^ BMDMs treated by different formulations (*n* = 5 independent samples, mean ± S.D.). Secretion of typical (E) pro-inflammatory cytokines (TNF-α, IL-1β, and IL-6) and (F) anti-inflammatory cytokines (IL-10) of LPS/INF-γ stimulated BMDMs following treatment with various siRNA formulations (*n* = 5 independent samples, mean ± S.D.). Statistical analyses were performed by one-way ANOVA with a subsequent Games–Howell post hoc test. *P < 0.05, **P < 0.01, and ***P < 0.001.

Collectively, these findings demonstrate that FW-LP@siIRF5 not only efficiently silenced IRF5 expression in LPS/INF-γ-stimulated BMDMs but also exhibited superior anti-inflammatory capabilities by polarizing inflammatory macrophages toward the anti-inflammatory M2-like phenotype, thereby highlighting the potential of FW-LP@siIRF5 for atherosclerosis treatment.

### In Vivo Pharmacokinetics of siRNA in Liposomal Formulations

To investigate the pharmacokinetics of siRNA in circulation, we intravenously injected various Cy5-labeled siIRF5 formulations (20 μg Cy5-siIRF5 per mouse) to plaque-bearing *ApoE* ^–/–^ mice that had been fed a high-fat diet (HFD) for 12 weeks. Blood samples were collected at predetermined points postinjection, and the fluorescence intensity of Cy5 was measured to monitor siRNA levels in the bloodstream. As shown in the blood decay curves, siRNA encapsulated in liposomal formulations (PEG-LP@Cy5-siIRF5, WRK-LP@Cy5-siIRF5, FA-LP@Cy5-siIRF5, and FW-LP@Cy5-siIRF5) exhibited sustained circulation, with 16–18% of the initial Cy5-siIRF5 signal still detectable in the blood at 8 h postinjection (Figure 4A). In stark contrast, free Cy5-siIRF5 degraded rapidly and cleared from the circulation, with nearly undetectable fluorescence signal as early as 1 h postinjection (Figure 4A). Interestingly, the addition of the WRK cell-penetrating peptide or the FA targeting ligand did not markedly alter the pharmacokinetics of the liposomal formulations. PEG-LP@Cy5-siIRF5 displayed similar blood retention profiles to those of WRK-LP@Cy5-siIRF5, FA-LP@Cy5-siIRF5, and FW-LP@Cy5-siIRF5 (Figure 4A), suggesting that these modifications did not significantly impact circulation half-life.

**Figure 4:**
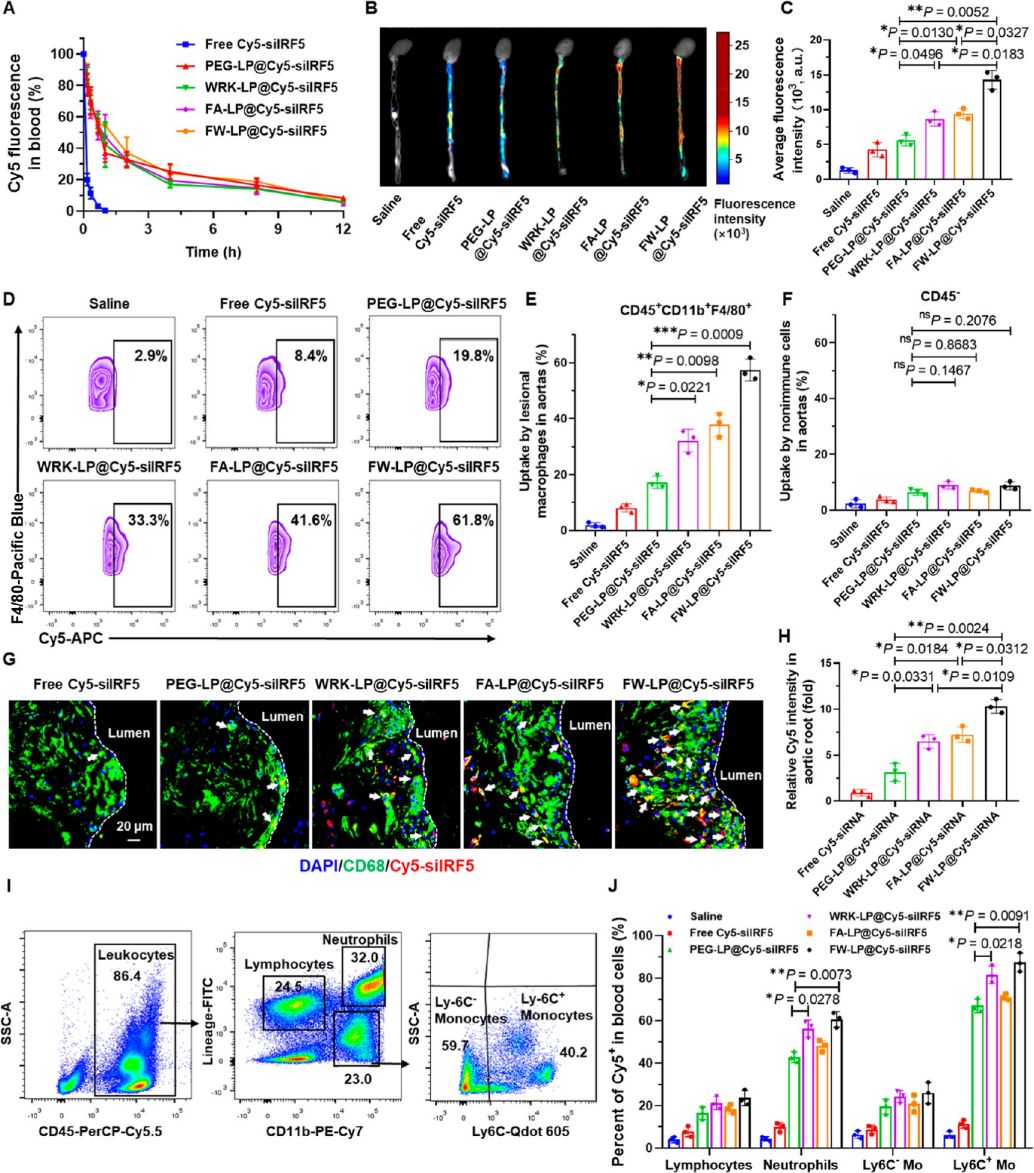
Evaluation of pharmacokinetics, biodistribution, plaque-targeting capability and, uptake mechanism of FW-LP@siIRF5 in *ApoE* ^–/–^ mice. (A) Pharmacokinetics of different formulations as indicated by time-dependent blood Cy5-siIRF5 concentration curves in *ApoE* ^–/–^ mice. Mice were injected intravenously with free Cy5-siIRF5 or various LPs@Cy5-siIRF5 formulations (including PEG-LP@Cy5-siIRF5, WRK-LP@Cy5-siIRF5, FA-LP@Cy5-siIRF5, and FW-LP@Cy5-siIRF5) at a dose of 20 μg Cy5-siIRF5 per mouse. Blood was collected at predetermined time points, and Cy5 fluorescence intensity was measured. The 0 h fluorescence intensity in each group was normalized to 100% (*n* = 3 biologically independent mice, mean ± S.D.). (B) Representative ex vivo fluorescence image of aortas harvested from plaque-bearing *ApoE* ^–/–^ mice 12 h after intravenous injection of various Cy5-siIRF5 formulations at a dose of 20 μg of Cy5-siIRF5 per mouse. (C) Quantification of Cy5 fluorescence intensity in aortas using ImageJ (*n* = 3 biologically independent mice, mean ± S.D.). (D–F) Flow cytometry-based quantification of Cy5 uptake in aortic cells 12 h postinjection. (D,E) Uptake in lesional macrophages (CD45^+^CD11b^+^F4/80^hi^) and (F) uptake in nonimmune cells (CD45^–^) in digested aortas obtained from atherosclerotic mice following treatment with different formulations at a dose of 20 μg of Cy5-siIRF5. Gating strategy is consistent with Figure S14. (*n* = 3 biologically independent mice, mean ± S.D.). (G) Confocal microscopy images illustrating colocalization of FW-LP@Cy5-siIRF5 (red) and CD68^+^ macrophages (green) in atherosclerotic plaques of the aortic roots. Nuclei were stained with DAPI (blue). White arrows indicate colocalization. Figure S11 also presents the corresponding original unmerged images. Scale bars = 20 μm. (H) Quantification of relative Cy5 fluorescence intensity in aortic root sections 12 after treatment, normalized to the free Cy5-siIRF5 group (*n* = 3 biologically independent mice, mean ± S.D.). (I) Gating strategy used for blood cell specificity by flow cytometry. Ly-6C^hi^ monocytes were identified as CD45^+^CD11b^hi^Lin^–/low^ Ly-6C^hi^ cells (with Lin^+^ defined as CD90.2^+^CD45R^+^ CD49b^+^NK1.1^+^Ly-6G^+^Ter119^+^). Neutrophils were identified as CD45^+^CD11b^hi^ Lin^hi^ cells. Ly-6C^low^ monocytes were identified as CD45^+^CD11b^hi^Lin^–/low^ Ly-6C^low^ cells. Lymphocytes were identified as CD45^+^CD11b^low^Lin^+^ cells. (J) Quantitative analysis of Cy5^+^ cells among blood cell subtypes (*n* = 3 biologically independent mice, mean ± S.D.). Statistical analyses were performed by one-way ANOVA with a subsequent Games–Howell post hoc test. (C, H), or Dunnett’s T3 post hoc test (E, F, and J). *P < 0.05, **P < 0.01, and ***P < 0.001, and ns denotes no significance.

These results demonstrated that liposomal encapsulation effectively enhances the stability and prolongs the circulation time of siRNA in vivo. This pharmacokinetic improvement is expected to facilitate enhanced accumulation of siRNA-loaded nanoparticles in atherosclerotic plaques, thereby contributing to improved therapeutic outcomes.^42^

### Plaque-Targeting Capability and Mechanism of FW-LP@Cy5-siRNA In Vivo

To evaluate the plaque-targeting capability of the liposomal delivery system, we intravenously injected various Cy5-labeled siIRF5 formulations into plaque-bearing *ApoE* ^–/–^ mice. Twelve h postinjection, aortas and major organs (kidneys, lungs, spleen, and liver) were harvested for ex vivo near-infrared fluorescence (NIRF) imaging (Figures 4B, S10). As shown in Figure 4B,C, FW-LP@Cy5-siIRF5 exhibited significantly higher Cy5 fluorescence in the aortas—approximately 1.7- and 1.6-fold greater than WRK-LP@Cy5-siIRF5 and FA-LP@Cy5-siIRF5, respectively. Both WRK-LP and FA-LP also demonstrated higher aortic accumulation than the nonmodified PEG-LP@Cy5-siIRF5 (Figure 4B,C).

Biodistribution analysis further revealed lower liver uptake of WRK- and FA-coated LPs@Cy5-siIRF5 compared to PEG-LP@Cy5-siIRF5 (Figure S10), supporting enhanced plaque accumulation of WRK-LP@Cy5-siIRF5 and FA-LP@Cy5-siIRF5. These NIRF imaging results collectively indicated that the WRK cell-penetrating peptide and the FA targeting ligand act synergistically to enhance FW-LP@Cy5-siIRF5 accumulation in atherosclerotic plaques. The increased accumulation is likely attributed to (1) WRK-mediated penetration through plaque-associated barriers, facilitating deeper infiltration;^31,32^ (2) FA-mediated binding to folate receptor-β (FA-β), which is overexpressed on lesional macrophages.^36,37^

To determine macrophage-specific uptake in atherosclerotic plaques, we performed flow cytometry and confocal microscopy on aortic tissues. Flow cytometry analysis demonstrated preferential uptake of Cy5-siRNA LPs by lesional macrophages (CD45^+^CD11b^+^F4/80^+^, Table S3), as compared with nonimmune cells (CD45^–^) in atherosclerotic lesions (Figure 4D–F). Among all formulations, FW-LP@Cy5-siIRF5 showed the highest macrophage uptake, exceeding both WRK-LP@Cy5-siIRF5 and FA-LP@Cy5-siIRF5. These findings were supported by confocal imaging, which revealed extensive colocalization of Cy5 signal (red) with CD68^+^ macrophages (green) in plaques (Figures 4G, S11). Quantification showed that WRK-, FA-, and FW-modified LPs achieved ∼1.5×, ∼1.9×, and ∼2.6× higher macrophage colocalization, respectively, compared to PEG-LP@Cy5-siIRF5 (Figure 4H). These findings were consistent with the flow cytometry data (Figure 4E); these results confirm that WRK and FA modifications significantly enhance both plaque penetration and macrophage targeting.

To further investigate biodistribution mechanisms, we examined blood cell interactions 12 h after FW-LP@Cy5-siIRF5 injection via flow cytometry on dissociated aortas (Figure 4I, and Table S4). Approximately 85% of Ly-6C^high^ monocytes and 60% of neutrophils internalized FW-LP@Cy5-siIRF5 (versus only ∼26% of Ly-6C^low^ monocytes and ∼23% of lymphocytes) (Figure 4J). In addition, WRK functionalization significantly boosted uptake by Ly-6C^high^ monocytes and neutrophils (Figure 4J), suggesting that, in addition to nanoparticles accumulating in plaques themselves, these inflammatory cells may act as circulating carriers that transport FW-LP@siIRF5 to atherosclerotic sites.^43,44^

### Antiatherosclerotic Effect of FW-LP@siIRF5 In Vivo

Encouraged by the efficient plaque-targeting capability of FW-LP@siIRF5, we subsequently evaluated its therapeutic efficacy in atherosclerotic plaque-bearing mice. Seven-week old *ApoE* ^–/–^ mice were first fed with a HFD for 8 weeks to establish atherosclerotic plaques. The atherosclerotic mice were then randomly assigned into four groups, and intravenously administered saline, PEG-LP@siIRF5, FW-LP@siIRF5, or FW-LP@siScr (20 μg siRNA per mouse) twice weekly for another 8 weeks while maintaining the HFD (Figure 5A). The PEG-LP@siIRF5 group served as a control to assess the benefits of the WRK cell-penetrating peptide and FA targeting ligand, while FW-LP@siScr served to confirm the therapeutic specificity of siIRF5.

**Figure 5:**
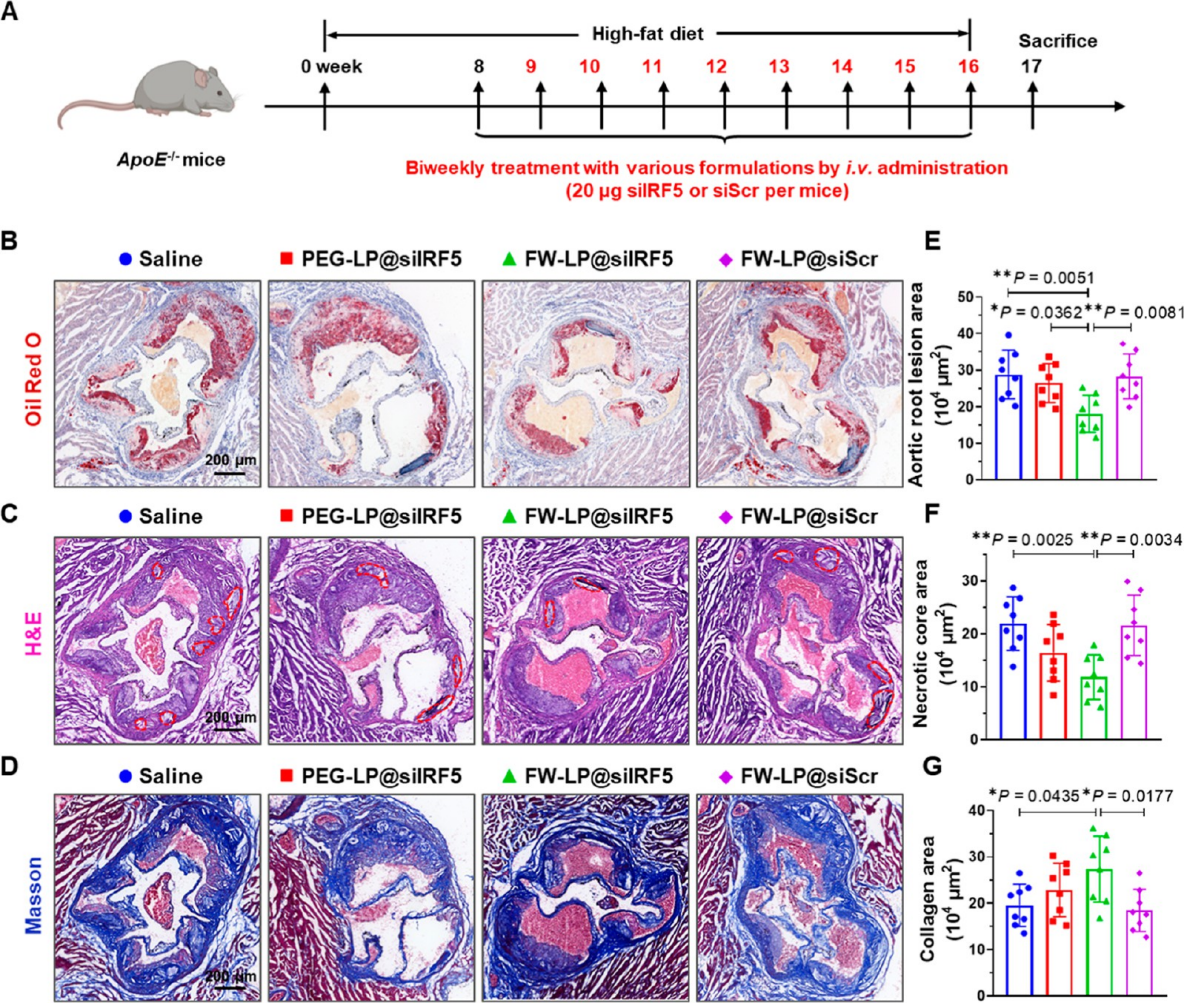
Antiatherosclerotic effect of FW-LP@siIRF5 in plaque-bearing *ApoE* ^–/–^ mice. (A) Schematic timeline of the siRNA treatment regimen. Seven-week-old *ApoE* ^–/–^ mice were fed a high-fat diet (HFD) for 8 weeks to induce advanced atherosclerosis. Mice were then randomly divided into four groups and intravenously administered with saline PEG-LP@siIRF5, FW-LP@siIRF5, and FW-PEG-LP@siScr at equivalent dosage of 20 μg IRF5 siRNA per mouse, twice weekly for an additional 8 weeks while maintaining on the HFD. One week after the final administration, mice were sacrificed, and blood and tissue samples were collected for molecular pathological assessments. (B–D) Representative histological images of aortic root sections stained with (B) ORO, (C) H&E, (D) Masson’s trichrome. Necrotic core areas were indicated by red dotted circles in (C). Scale bars, 200 μm. (E–G) Quantitative analysis of aortic root sections, including (E) lesion area, (F) necrotic core area, (G) collagen area in aortic root sections by Image-Pro Plus 6.0 software (*n* = 8 biologically independent mice, mean ± S.D.). Statistical analyses were performed by one-way ANOVA with a subsequent Tukey’s post hoc test. *P < 0.05, and **P < 0.01. Cartoon mouse in (A) was created with BioRender.com.

One week after the final administration, whole aortas and aortic roots were harvested and subjected to histological analyses to quantify lesion burden and assess plaque stability. Oil Red O (ORO) staining was used to visualize and quantify atherosclerotic lesions, with red-stained areas indicating lipid-rich plaques (Figures 5B, S12A).^6,45,46^ Both ORO-stained images and corresponding quantitative analysis revealed that FW-LP@siIRF5 treatment significantly reduced lesion areas in the entire aorta (Figure S12A,B) and aortic roots (Figures 5B,E, and S10A) outperforming all other treatment groups. In contrast, atherosclerotic mice treated with PEG-LP@siIRF5 displayed only moderate reductions in plaque aortas (Figure S12A,B) and aortic roots (Figures 5B,E, and S13A). These results highlight the synergistic role of WRK cell-penetrating peptide and FA targeting ligand in enhancing plaque-targeted siRNA delivery, while validating the antiatherosclerotic potential of siIRF5 nanoimmunotherapy in treating atherosclerosis.

Plaque stability was further evaluated by histological assessment with hematoxylin and eosin (H&E) and Masson’s trichrome staining. H&E staining and quantitative analysis revealed the largest lipid-rich necrotic cores (outlined by red dashed line) in the saline and FW-LP@siScr cohorts (Figures 5C, F, and S10B). Treatment with PEG-LP@siIRF5 slightly reduced necrotic core areas within plaques (Figures 5C, F and S10B), likely due to a combination of siIRF5 therapeutic effect, prolonged circulation time, and moderate plaque accumulation through the leaky vascular. Notably, FW-LP@siIRF5 significantly diminished necrotic core size, likely owing to efficient macrophage-targeted delivery and effective IRF5 silencing within plaques (Figures 5C, F, and S10B).

Masson’s trichrome staining was used to assess collagen content (blue)—a key indicator of plaque stability.^6,45^ FW-LP@siIRF5-treated mice exhibited markedly increased collagen deposition (blue-stained areas), indicating stronger fibrous caps and enhanced plaque stability (Figures 5D, G, and S10C). Enhanced collagen content reduces the risk of plaque rupture and subsequent thrombotic events.

Consequently, these results demonstrate that FW-LP@siIRF5 significantly reduces plaque burden, decreases necrotic core size, and increases collagen content in atherosclerotic lesions. These findings highlight the therapeutic potential of FW-LP@siIRF5 as a macrophage-targeted nanotherapeutic for effective treatment of atherosclerosis.

### IRF5 Gene Silencing Effect and Antiatherosclerotic Mechanism of FW-LP@siIRF5 In Vivo

After confirming the FW-LP@siIRF5′s antiatherosclerotic efficacy, we next assessed its gene-silencing efficiency in vivo and investigated the underlying therapeutic mechanism by analyzing lesional macrophage phenotypes in *ApoE* ^–/–^ mice. We first isolated plaque-bearing whole aortas from treated *ApoE* ^–/–^ mice and prepared single-cell suspensions via homogenization and enzymatic digestion. These suspensions were stained with a flow cytometry antibody panel (Table S5, Figure S14: anti-CD45, anti-CD11b, anti-F4/80, and anti-IRF5) to analyze IRF5 expression in lesional macrophages (Figure 6A,B). The results revealed that the elevated IRF5 expression observed in lesional macrophages from saline-treated mice was markedly reduced in the FW-LP@siIRF5-treated cohort. Notably, FW-LP@siIRF5 achieved greater IRF5 knockdown than either PEG-LP@siIRF5 or FW-LP@siScr controls (Figure 6B), underscoring the pivotal roles of the WRK cell-penetrating peptide and FA targeting ligand in enhancing macrophage-specific delivery. These data validate that FW-LP@siIRF5 effectively downregulates IRF5 expression in lesional macrophages, supporting IRF5 as a promising therapeutic target for atherosclerosis.

**Figure 6:**
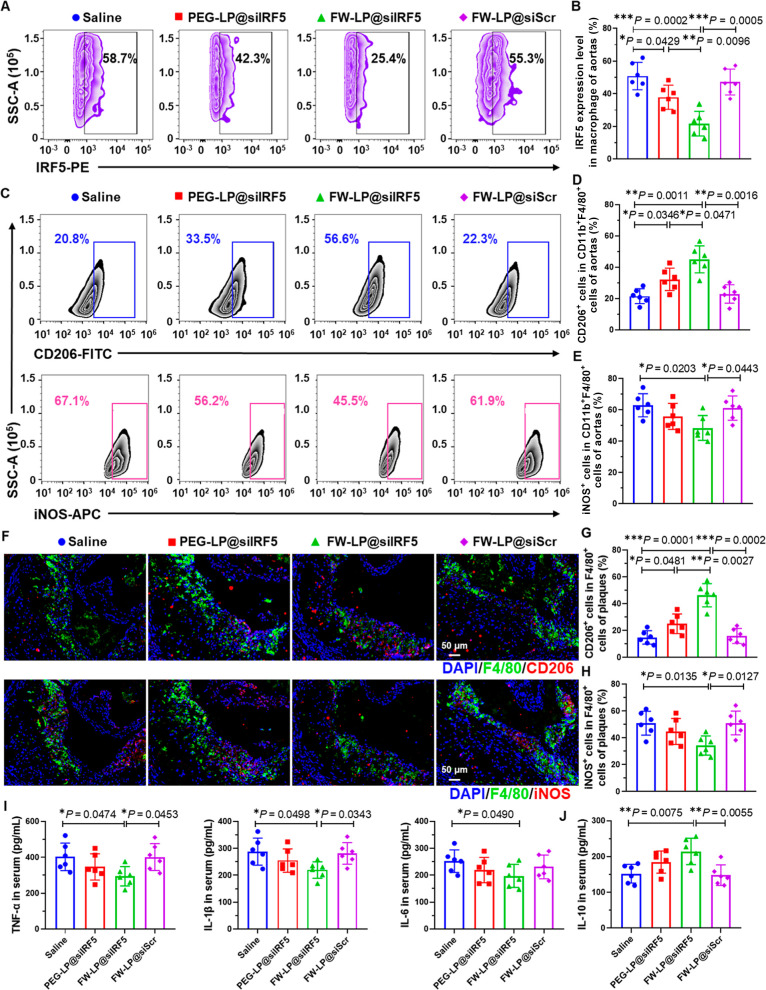
In vivo IRF5 gene silencing and antiatherosclerotic mechanisms of FW-LP@siIRF5. (A) Representative flow cytometry contour plot and (B) quantitative analysis of IRF5 expression in lesional macrophage (CD45^+^CD11b^+^F4/80^+^) isolated from digested aortas of atherosclerotic mice treated by different formulations (*n* = 6 biologically independent mice, mean ± S.D.). Gating strategy is detailed in Figure S14. (C–E) Flow cytometry analysis of M2-like (CD206^+^) and M1-like (iNOS^+^) macrophages among lesional macrophages (CD45^+^CD11b^+^F4/80^+^). Gating strategy in provided in Figure S14. (C) Representative flow cytometry histograms and quantification of (D) CD206^+^ cells and (E) iNOS^+^ cells in lesional macrophages (CD45^+^CD11b^+^F4/80^+^) from digested aortas. (*n* = 6 biologically independent mice, mean ± S.D.). (F) Representative confocal microscopy images and quantitative analysis of (G) CD206^+^ and (H) iNOS^+^ cells (red) in lesional macrophages (green) in cross sections of the aortic root from plaque-bearing *ApoE* ^–/–^ mice after different treatments (*n* = 6 biologically independent mice, mean ± S.D.). Blue: DAPI staining for cell nuclei. Figure S15 also presents the corresponding original unmerged images. Scale bars, 50 μm. (I) Serum concentrations of pro-inflammatory cytokines (TNF-α, IL-1β, and IL-6) and (J) anti-inflammatory cytokine IL-10 in atherosclerotic mice receiving different treatments (*n* = 6 biologically independent mice, mean ± S.D.). Statistical analyses were performed by one-way ANOVA with a subsequent Games–Howell post hoc test., or using an unpaired two-tailed *t* test (I). *P < 0.05, **P < 0.01, ***P < 0.001.

To explore the effect of FW-LP@siIRF5 knockdown on macrophage polarization, we examined the expression of M2-like (CD206^+^) and M1-like (iNOS^+^) markers in CD45^+^CD11b^+^F4/80^+^ lesional macrophages from digested aortas using flow cytometry (Figure 6C–E, Table S5, and Figure S14). FW-LP@siIRF5 treatment significantly increased the proportion of CD206^+^ cells (Figure 6C,D) while reducing iNOS^+^ macrophages (Figure 6C, E), indicating successful repolarization of lesional macrophages toward an anti-inflammatory M2-like phenotype and suppression of the pro-inflammatory M1 phenotype.

These findings were further corroborated by immunofluorescence analysis of aortic root tissue sections. FW-LP@siIRF5-treated mice exhibited the highest levels of F4/80^+^CD206^+^ (M2-like) macrophages and the lowest levels of F4/80^+^iNOS^+^ (M1-like) macrophages (Figures 6F–H, and S15A,B), consistent with the flow cytometry results. These results indicate that FW-LP@siIRF5 reprogrammed lesional macrophages by promoting M2-like, anti-inflammatory characteristics and reducing M1-like, pro-inflammatory phenotype in the atherosclerotic environment.

To evaluate the systemic and local anti-inflammatory effects of this macrophage repolarization, we quantified pro-inflammatory cytokines including TNF-α, IL-1β, and IL-6, together with the anti-inflammatory cytokine IL-10, in both aortic tissues and serum. FW-LP@siIRF5 treatment markedly attenuated TNF-α, IL-1β, and IL-6 levels while substantially elevating IL-10 expression in both serum (Figures 6I,J) and aortic tissue (Figure S15C,D). These results highlight the potent inflammation-resolving activity of FW-LP@siIRF5.

Collectively, the superior antiatherosclerotic efficacy of FW-LP@siIRF5 arises from its ability to silence IRF5 expression selectively in lesional macrophages, which in turn promotes macrophage repolarization toward an M2-like anti-inflammatory phenotype. This immunomodulatory shift leads to inflammation resolution, reduced plaque burden, and enhanced plaque stability.

### IRF5 Silencing and Antiatherosclerotic Effect and Mechanism of FW-LP@siIRF5 in an Advanced Accelerated Plaque Rupture Model

To further evaluate the gene-silencing efficacy of IRF5 and its impact on atherosclerotic progression in multiple atherosclerotic models, we employed an accelerated model of plaque destabilization and rupture in the brachiocephalic arteries, induced by angiotensin II infusion in *ApoE* ^–/–^ mice^6,47^ (Figure 7A). Plaque-bearing mice received biweekly injections for 4 weeks with saline, PEG-LP@siIRF5, FW-LP@siIRF5, or FW-LP@siScr (20 μg siRNA per mouse). One day after the final dose, brachiocephalic arteries were harvested to assess IRF5 silencing efficiency in lesional macrophages by immunofluorescence staining. Additionally, plaque stability was evaluated using H&E and elastica van Gieson (EVG) staining assays. Immunofluorescence analysis revealed that IRF5 expression was markedly reduced in lesional macrophages within brachiocephalic artery plaques from FW-LP@siIRF5-treated mice compared with the other groups (Figures 7B,C, S16), consistent with the results from the chronic atherosclerosis model (Figure 6A,B). These results demonstrated that FW-LP@siIRF5 consistently retains robust IRF5-silencing activity across multiple animal models of atherosclerosis. Additionally, H&E staining demonstrated a significant reduction in necrotic core area in plaque of the FW-LP@siIRF5 cohort (13.9 × 10^4^ μm^2^) compared with saline (25.4 × 10^4^ μm^2^), PEG-LP@siIRF5 (22.6 × 10^4^ μm^2^) and FW-LP@siScr (24.9 × 10^4^ μm^2^) cohorts (Figure 7D–F), indicating that FW-LP@siIRF5 effectively suppressed necrotic core formation. Furthermore, EVG staining revealed a significant decrease in disrupted or buried fibrous caps and an increase in fibrous cap thickness in the FW-LP@siIRF5 group (Figure 7D, G,H), demonstrating enhanced plaque stability and reduced vulnerability to rupture.^47^

**Figure 7:**
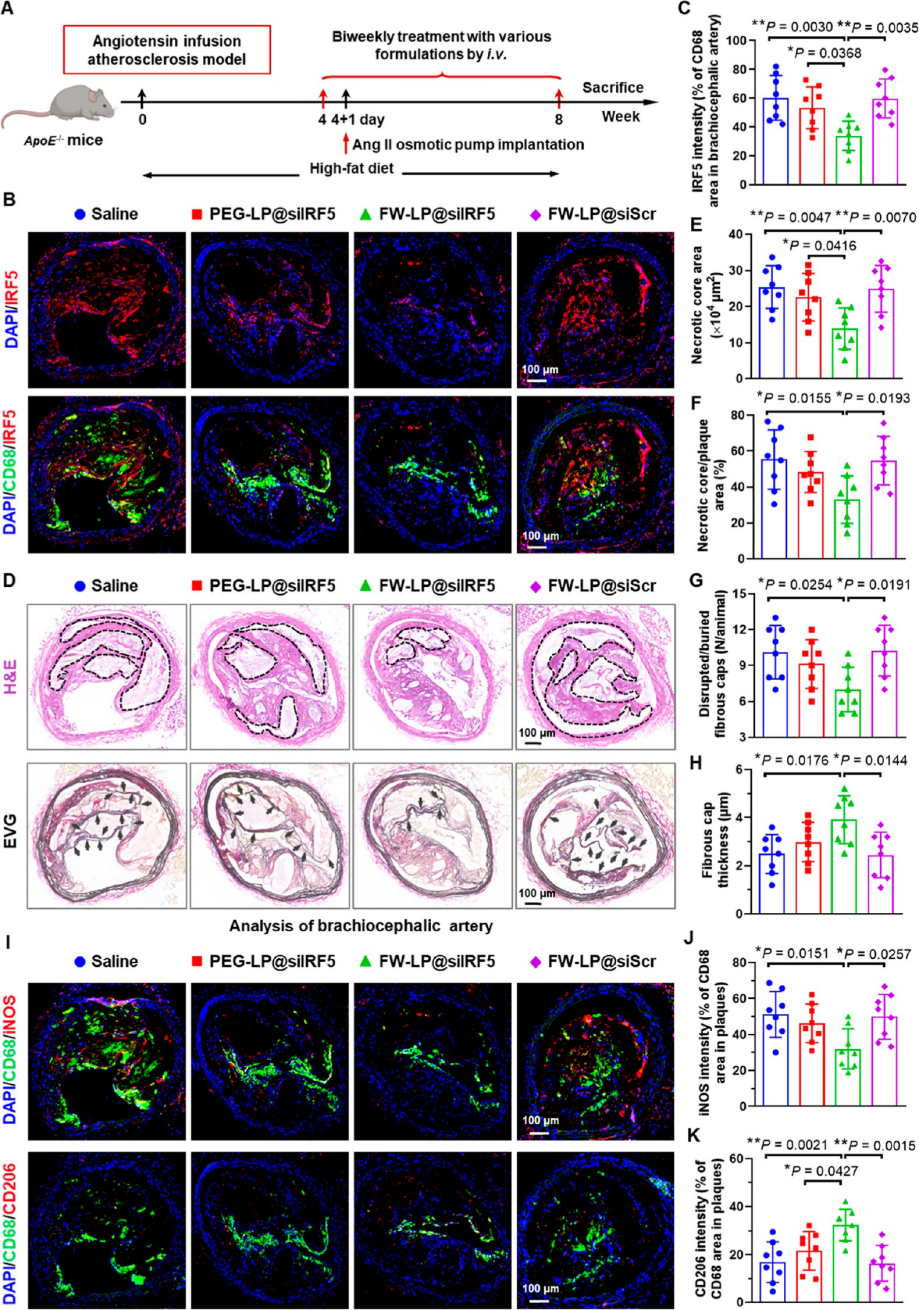
IRF5 in lesional macrophages, reprograms macrophage phenotype, and prevents atherosclerotic plaque destabilization and rupture in angiotensin-infused plaque-bearing *ApoE* ^–/–^ mice. (A) Schematic illustration of the angiotensin-induced atherosclerotic plaque rupture model and siRNA treatment protocol. Twelve-week-old *ApoE* ^–/–^ mice were fed a HFD for 4 weeks, then infused with angiotensin II and treated with various siRNA formulations for another 4 weeks while continuing on the HFD. (B) Representative immunofluorescence images of atherosclerotic lesions within brachiocephalic artery sections showing IRF5^+^ cells (IRF5, red), macrophages (CD68, green), and nuclei (DPAI, blue). Scale bars = 100 μm. (C) Quantification of IRF5 expression in lesional macrophages in brachiocephalic artery sections across treatment groups (*n* = 8 biologically independent mice). (D) H&E and elastica van Gieson (EVG) staining of plaques showing atherosclerotic plaques in brachiocephalic artery. The black dotted lines in H&E staining delineate the necrotic area. Arrowheads in EVG-stained sections highlight disrupted or buried fibrous caps within the lesions. Scale bars = 100 μm. (E,F) Quantification of necrotic area and necrotic core/plaque area in brachiocephalic artery sections using H&E staining (*n* = 8 biologically independent mice). (G,H) Quantification of (G) the number of disrupted/buried fibrous caps and (H) fibrous cap thickness in atherosclerotic plaques of brachiocephalic artery sections using EVG staining (*n* = 8 biologically independent mice). (I) Representative immunofluorescence images of atherosclerotic lesions within brachiocephalic artery sections depicting INOS^+^ or CD206^+^ cells (red), macrophages (CD68, green), and nuclei (DPAI, blue). The corresponding original unmerged images are also presented in Figure S17A and B. Scale bars = 100 μm. (J,K) Quantification of (J) iNOS and (K) CD206 expression in lesional macrophages in brachiocephalic artery sections after different treatments (*n* = 8 biologically independent mice). Data were analyzed using one-way ANOVA with a Tukey post hoc test. All graphs present data as mean ± SD *P < 0.05, **P < 0.01, and ***P < 0.001, while *P* > 0.05 denotes no significance. Cartoon mouse in (A) was created with BioRender.com.

Moreover, in vivo macrophage phenotype analysis revealed that FW-LP@siIRF5 effectively repolarized lesional macrophages toward an anti-inflammatory M2-like phenotype, while simultaneously suppressing the pro-inflammatory M1-like phenotype within plaques (Figures 7I–K, and S17A,B). This macrophage repolarization effect was in line with the phenotypic shifts observed in the chronic atherosclerosis model (Figure 6F–H), suggesting a consistent mechanism contributing to the therapeutic efficacy of FW-LP@siIRF5. To further validate the functional impact of macrophage reprogramming on atherosclerotic inflammation, we examined the expression of pro-inflammatory cytokines, including TNF-α, IL-1β, and IL-6, together with the anti-inflammatory cytokine IL-10 in both local plaque tissues (Figure S17C) and systemic serum (Figure S17D). FW-LP@siIRF5 treatment markedly reduced the levels of pro-inflammatory cytokines while significantly elevating IL-10. These results demonstrate a robust inflammation-resolving capacity of FW-LP@siIRF5 in the angiotensin II–infused atherosclerosis model, mirroring the anti-inflammatory profile observed in the chronic model (Figures S15C,D, and 6I,J).

Taken together, these findings establish that FW-LP@siIRF5 not only suppresses plaque progression and rupture but also promotes resolution of vascular inflammation resolution. By efficiently silencing IRF5 and reprogramming lesional macrophages, FW-LP@siIRF5 exhibits potent and reproducible antiatherosclerotic efficacy across multiple murine models of atherosclerosis.

### In Vivo Biosafety of FW-LP@siIRF5

Given the critical importance of biocompatibility for the clinical translation of innovative nanotherapeutics, we systemically evaluated the long-term in vivo biosafety of FW-LP@siIRF5 nanoparticles in *ApoE* ^–/–^ mice after repeated administration in two atherosclerosis models. In the chronic atherosclerosis model, body weight curves (Figure S18), organ histology (Figure 8A), blood chemistry profiles (Figure 8B), and hepatic and renal function biomarkers (Figure 8C) in the treatment group were comparable to those in saline-treated controls, indicating tolerability during prolonged administration. Additionally, systemic metabolic indices, including total cholesterol, triglycerides, fasting glucose, and low-density lipoprotein (Figure 8D), indicating that the therapeutic benefits of FW-LP@siIRF5 were not attributable to cholesterol-lowering effects—a conventional mechanism in atherosclerosis therapy. Furthermore, consistent with the safety profile observed in the chronic model, FW-LP@siIRF5 nanotherapeutics also did not induce any detectable toxicities in angiotensin-infused atherosclerotic mice after 4 weeks of treatment (Figure S19). Together, these results demonstrate that FW-LP@siIRF5 nanotherapeutics exert potent antiatherosclerotic effects through IRF5 silencing-mediated macrophage reprogramming while maintaining long-term biocompatibility, underscoring its translational potential as a therapeutic strategy for chronic vascular diseases.

**Figure 8:**
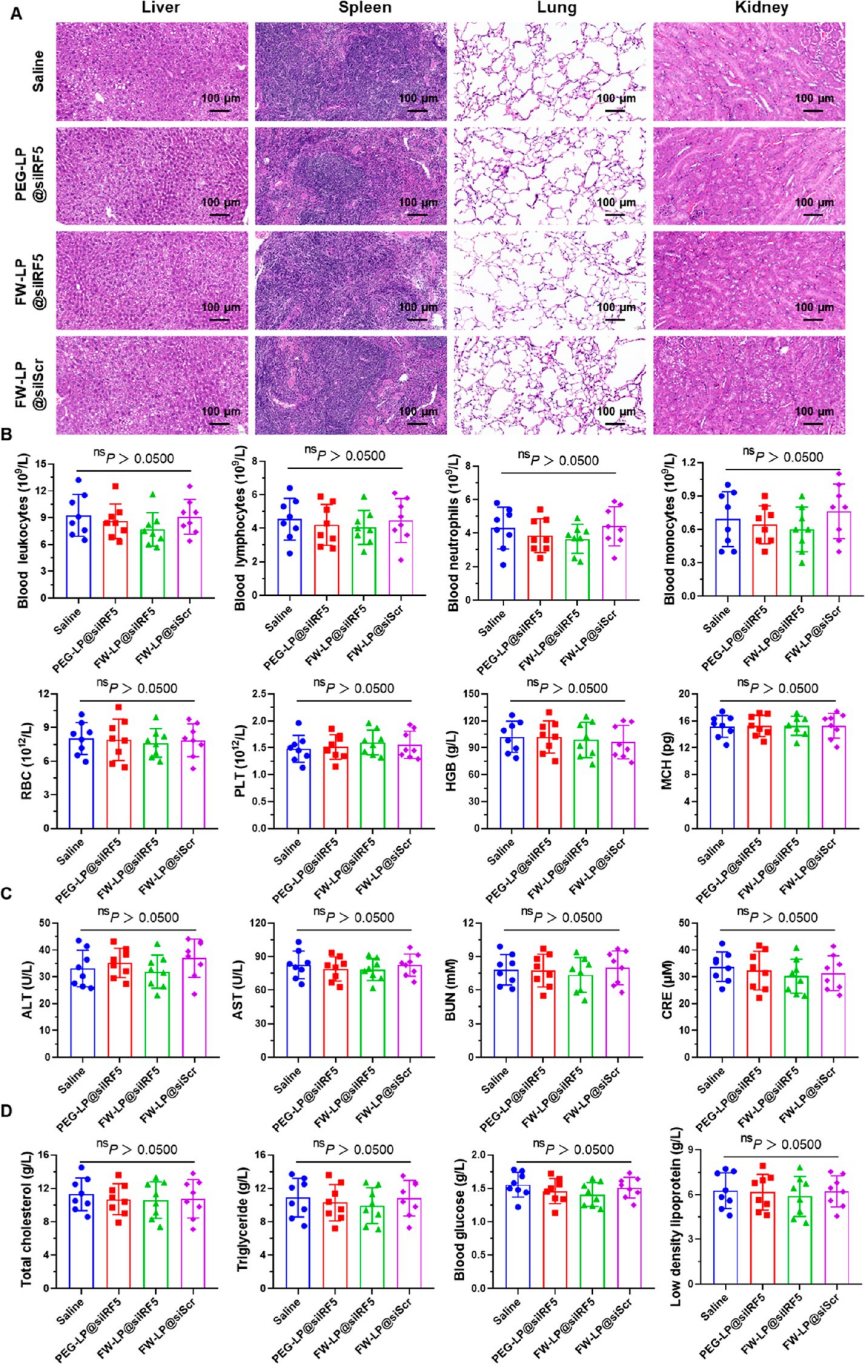
In vivo evaluation of biocompatibility of atherosclerotic *ApoE* ^–/–^ mice treated with various therapies. Eight-week HFD-fed *ApoE* ^–/–^ were injected intravenously with saline or differenent siRNA nanomedicines (PEG-LP@siIRF5, FW-LP@siIRF5, and FW-LP@siScr; siRNA, 20 μg per mouse) twice a week for an additional 8 weeks, while being maintained on the HFD. Mice were euthanized 1 week after the final injection, and organs and blood were collected for histopathological analysis, as well as for the analysis of blood biochemistry and metabolic parameters. (A) Representative H&E-stained images of major organs of atherosclerotic *ApoE* ^–/–^ mice after various treatments. Scale bars, 100 μm. (B) Hematological parameters, including leukocytes, lymphocytes, neutrophils, monocytes, red blood cell count (RBC), platelet count (PLT), hemoglobin (HGB), and mean corpuscular hemoglobin (MCH) from atherosclerotic *ApoE* ^–/–^ mice, as described in Figure 5 (*n* = 8 biologically independent mice). (C) Serum concentrations of liver function biomarkers (ALT and AST) and kidney function biomarkers (BUN and CRE) in the same cohort (*n* = 8 biologically independent mice). (D) Serum levels of metabolic indices, including total cholesterol, triglycerides, fasting glucose, and low-density lipoprotein (*n* = 8 biologically independent mice). No significant differences were observed across treatment groups for these metabolic parameters, indicating that FW-LP@siIRF5 treatment does not disrupt cholesterol or glucose homeostasis in vivo. Data are presented as mean ± SD. Group differences were analyzed by one-way ANOVA followed by Tukey’s post hoc test (n.s., not significant).

Furthermore, we systematically evaluated the in vivo biosafety of both empty FW-LP and FW-LP@siIRF5 in wild-type C57BL/6J mice using a combination of hematological, blood biochemical, and histological analyses. Specifically, a total of 18 mice were randomly divided into three groups and intravenously administered with saline, empty FW-LP, or FW-LP@siIRF5 every other day over a 14 day period. At 48 h following the last administration, the mice were were sacrificed, and peripheral blood together with major organs, including the heart, liver, spleen, lung, and kidney, were harvested for comprehensive biosafety assessment. Histological analysis of H&E-stained tissue sections showed no evident pathological abnormalities or organ damage across all treatment groups (Figure S20A). Blood biochemical assays (Figure S20B) showed no significant differences in hepatic function biomarkers (alanine transaminase (ALT) and aspartate aminotransferase (AST)) or renal function biomarkers (blood urea nitrogen (BUN) and creatinine (CRE)) between treated and control groups, indicating preserved liver and kidney function. Furthermore, complete blood count analysis (Figure S20C) showed no significant alterations in hematological parameters, including leukocytes, lymphocytes, neutrophils, monocytes, red blood cells (RBC), hemoglobin (HGB), platelets (PLT), and mean corpuscular hemoglobin (MCH), suggesting no hematotoxicity or adverse effects on the immune system. Moreover, serum inflammatory cytokine levels (TNF-α, IL-1β, and IL-6) remained unchanged across the groups (Figure S20D), and no significant differences in body weight were observed during the treatment period (Figure S20E), further supporting the absence of systemic immune activation or toxicity. Finally, to determine whether our nanoimmunotherapy compromises host defense against infection, we performed an additional acute Methicillin-resistant *Staphylococcus aureus* (MRSA) infection model via intravenous challenge^48,49^ The inflammatory responses in treated mice were evaluated by measuring serum pro-inflammatory cytokines and chemokine (TNF-α, IL-6, IL-1β, and Cxcl2), the anti-inflammatory cytokine IL-10, and monitoring body-weight changes. As shown in Figure S21, cytokine and chemokine levels and body-weight trajectories were comparable between the FW-LP@siIRF5-treated group and the saline control group, with no significant differences observed. These findings indicate that the treatment does not impair systemic inflammatory responses in this acute infection setting, suggesting that our nanoimmunotherapy does not induce general immunosuppression.

Together, these findings confirm that both empty FW-LP and FW-LP@siIRF5 are well-tolerated in vivo, inducing minimal immune response and exhibiting no overt toxicity. The biocompatibility and low immunogenicity of FW-LP@siIRF5 underscore its promise as a safe and effective nanoplatform for the long-term treatment of chronic vascular diseases, such as atherosclerosis.

## Discussion

Lesional macrophages play a critical role in atherosclerosis progression by inducing chronic inflammation in both systemic and lesional environments.^1,3,6^ Therefore, targeting specific signaling pathways in lesional macrophages and regulating macrophage-associated inflammatory processes have important implications for the regression of atherosclerosis. Remarkably, the transcription factor interferon regulatory factor 5 (IRF5) has been recently demonstrated to be strongly expressed in lesional macrophage of advanced human and mouse atherosclerotic plaques. IRF5 drives atherogenesis and persistent inflammation by polarizing macrophages toward the pro-inflammatory M1 phenotype, leading to the up-regulation of inflammation-associated cytokines and signaling pathways.^16–19^ Yet, targeting IRF5 for atherosclerotic cardiovascular disease therapy remains elusive. RNA interference (RNAi) is a potent technology capable of specifically silencing aberrant endogenous genes using siRNA, showing significant promise in the treatment of cardiovascular diseases.^23,24^ Recent advances have led to the development of siRNA-based strategies targeting key regulators of lipid metabolism in atherosclerosis. Notably, inclisiran—a GalNAc-modified siRNA targeting proprotein convertase subtilisin/kexin type 9 (PCSK9)—has shown durable LDL-C reduction with a favorable safety profile and is now approved for clinical use in hypercholesterolemia. This clinical success underscores the feasibility and translational potential of siRNA therapeutics in cardiovascular disease.^24^ However, PCSK9-targeting therapies primarily act by lowering systemic lipid levels and do not directly address immune dysregulation within atherosclerotic plaques. In contrast, our study plans to directly target IRF5, a transcription factor critically involved in driving macrophage-mediated inflammation in advanced plaques. By reprogramming lesional macrophages phenotype, our siIRF5-based nanoimmunotherapy addresses local inflammatory pathology—an aspect largely unaddressed by existing lipid-lowering or systemic anti-inflammatory regimens.

Previous preclinical studies have investigated siRNA strategies against inflammatory mediators,^50–52^ such as NF-κB, CCR2, and NLRP3, often using passive delivery mechanisms. While mechanistically compelling, these approaches have been constrained by off-target effects, poor plaque localization, and potential immunogenicity. Importantly, many of these strategies lack macrophage specificity, further limiting their therapeutic efficacy in modulating lesion-resident immune cells. Therefore, the development of a macrophage-targeted siRNA therapeutic capable of efficiently silencing IRF5 represents a promising and disease-relevant strategy for modulating intraplaque inflammation and halting atherosclerosis progression.

In this study, we developed an innovative FW-LP@siIRF5 siRNA liposomal nanoplatform to effectively inhibit the progression of atherosclerosis by silencing IRF5 expression in atherosclerotic lesional macrophages (Figure 1). The key design of our study was the construction of PEGylated DOTAP-based cationic liposomes to encapsulate siIRF5. The advantages of these PEGylated cationic liposomes are as follows: (i) The biocompatible DOTAP lipids exhibit a favorable siRNA loading capacity (Figure S3B) attributed to strong electrostatic interactions between the positively charged DOTAP in the nanoparticles and the negatively charged phosphate backbone of siRNA, as well as physical confinement of siRNA within the liposomal interior.^26,53^ (ii) The unique structure of the liposome protects the siRNA from nuclease degradation, ensuring its stability in the presence of serum nucleases (Figure S4).^54^ (iii) The lipid-PEG coating on the surface of the liposomes helps evade recognition by the mononuclear phagocyte system (MPS),^27–29^ prolonging the circulation lifetime of siRNA in the bloodstream (Figure 4A) and increasing its accumulation in atherosclerotic plaques (Figure 4B,C).^55^

To further enhance the delivery efficiency of siRNA and specifically target lesional macrophages within atherosclerotic plaques, we incorporated a cell-penetrating peptide (WRK) and a macrophage-targeting peptide (FA, folic acid) onto the lipid-PEG layer of the cationic lipids. Our results showed that the WRK cell-penetrating peptide and FA targeting ligand synergistically enhanced the accumulation of FW-LP@Cy5-siIRF5 in aortic tissues and lesional macrophages (Figures 4B–H, S11). This enhanced accumulation can likely be attributed to three key factors: (i) the WRK peptide can effectively penetrate the barriers of aortic plaques and infiltrate deeper layers to interact with immune cells,^31,32,34^ thereby increasing the presence of WRK-coated LPs@Cy5-siIRF5 in aortic plaques; (ii) the FA targeting ligand specifically binds to the overexpressed folic acid receptor on lesional macrophages within plaques,^36,37^ further enhancing the accumulation of FA-coated LPs@Cy5-siIRF5 in the aorta; (iii) after entering the bloodstream, WRK promotes uptake of the particles by Ly-6C^high^ monocytes and neutrophils (Figure 4I,J), which then migrate to sites of vascular inflammation.^43,44,56^ Together, these factors ensure the promise of FW-LP as nanoparticle-mediated immunotherapy in atherosclerosis, effectively delivering IRF5 siRNA to silence pro-atherosclerotic gene expression in lesional macrophages.

To enhance the translational significance of our study, we applied a dual-model strategy by combining a lipid-driven advanced plaque model under chronic inflammation with an accelerated plaque rupture model. This approach allowed us to rigorously assess the IRF5-silencing capacity and antiatherosclerotic effects of FW-LP@siIRF5 nano-immunotherapeutics under complementary pathological conditions. Our findings demonstrated that FW-LP@siIRF5 consistently reduced IRF5 expression in lesional macrophages of atherosclerotic plaques (Figures 6A,B, and 7B,C), repolarized inflammatory macrophages toward an anti-inflammatory M2-like phenotype (Figures 6C–H, and 7I–K), and promoted inflammation resolution (Figures 6I,J, and S17C,D) in two atherosclerosis models. These reproducible and model-independent outcomes highlight the robustness of this therapeutic strategy.

Consequently, FW-LP@siIRF5 inhibited atherosclerosis progression by reducing plaque burden and enhancing plaque stability (Figures 5 and 7), without altering plasma cholesterol levels, avoiding the repeated dosing and systemic immunosuppression risks inherent to single-cytokine neutralization strategies such as IL-1β monoclonal antibodies.^57^ Importantly, biosafety evaluation confirmed long-term tolerability, with no infection-related discomfort or significant off-target toxicity (Figures 8, and S18–S21). This favorable safety profile is attributed to both the intrinsic biocompatibility of the nanoparticles and their efficient accumulation within lesional macrophages, further supporting their translational potential.

Mechanistically, our dual-targeted nanoplatform integrates PEGylated cationic liposomes with WRK and FA ligands, enabling selective uptake by lesional macrophages while minimizing systemic exposure. This design addresses key limitations of conventional siRNA delivery, including rapid degradation, inefficient endosomal escape, and nonspecific biodistribution. By targeting the clinically relevant transcription factor IRF5 in a cell-specific manner, FW-LP@siIRF5 represents a promising and adaptable strategy for treating atherosclerosis and other macrophage-driven inflammatory diseases.

### Study Limitations

While our study introduces a promising siRNA-based nanotherapeutic strategy for targeting atherosclerosis-promoting genes in plaque macrophages, further investigations are needed to extend these findings across additional disease models and to optimize translational relevance. Moreover, our current validation is limited to the male *ApoE* ^–/–^ mouse model. Future studies utilizing complementary atherosclerosis models and sex—such as *LDL* ^–/–^ mice, and female *LDL* ^–/–^ and *ApoE* ^–/–^ mice—will be essential to assess model dependence and generalizability of our nanotherapeutic strategy. In addition, our research is currently limited to small animal models, which, despite offering valuable mechanistic insights, only moderately replicate the anatomical, physiological, and immunological characteristics of human atherosclerosis. To bridge this gap, future investigations employing large animal models—such as pigs with experimentally induced atherosclerosis—will be critical to further evaluate the in vivo therapeutic efficacy, safety, and biodistribution of the nanotherapeutic platform. These efforts will not only enrich the scientific robustness of our findings but also provide crucial data to support the clinical translation and feasibility of lesional macrophage-targeted RNAi therapies for advanced atherosclerosis.

## Conclusion

In summary, our study introduces targeted-peptide-modified siRNA nano-immunotherapeutics for atherosclerosis treatment by efficiently silencing IRF5 gene in atherosclerotic lesional macrophages to reprogramming phenotype. This study not only introduces an immunotherapeutic strategy for atherosclerosis treatment but also underscores the potential of targeted nucleic acid drug delivery systems for other macrophage-associated inflammatory diseases.

## Experimental Section

### Materials

Cholesterol (Chol), Chol-PEG_2K_–COOH, 1,2-Dioleoyl-3-trimethylammonium-propane (DOTAP), mPEG_2k_-chol, and Chol-PEG_2K_–OH were purchased from Ponsure (Shanghai, China). The undecorated cyclic peptide WRWRWRWRWK (W5R4K, WRK) containing tryptophan (W), arginine (R), and lysine (K) were purchased from Paihetaide Pharmaceutical Biotechnology (Zhengzhou, China). The undecorated folic acid were purchased from BOC Sciences (USA). All siRNAs were synthesized by Tsingke Biotechnologies (Beijing, China), and their sequences are as follows: (i) IRF5 (interferon regulatory factor 5): 5′-CUG CAG AGA AUA ACC CUG A-dTdT-3′ (sense) and 5′-UCA GGG UUA UUC UCU GCA G-dTdT-3′ (antisense); (ii) scramble: 5′-CUU ACG CUG AGU ACU UCG AdTdT-3′ (sense) and 5′-UCG AAG UAC UCA GCG UAA GdTdT-3′ (antisense); (iii) Cy5-IRF5:5′-Cy5-CUG CAG AGA AUA ACC CUG A-dTdT-3′ (sense) and 5′-UCA GGG UUA UUC UCU GCA G-dTdT-3′ (antisense). The selected siIRF5 sequence, proven effective in previous studies, demonstrated superior gene-silencing efficacy among several sequences.^18^ Cy5 was incorporated into the 5′-end of the sense strand of the siIRF5 sequence to generate Cy5-labeled siIRF5. All flow-cytometry antibodies were obtained from BD Biosciences, BioLegend, or Cell Signaling Technology (CST), as summarized in Tables S3, S4, and S5. All mouse glycoprotein ELISA kits (TNF-α, IL-Iβ, IL-6, and IL-10) were purchased from Solarbio (Beijing, China).

### Cell Culture

Bone marrow-derived macrophages (BMDMs) were generated from progenitor cells flushed from the tibias and femurs of 20 week-old C57BL/6J mice according to standard procedures.^23^ All cells were then cultured in T-75 flasks with vented caps. The cells were maintained in complete Dulbecco’s Modified Eagle Medium (DMEM)-high glucose (4.5 g/L) supplemented with 10% heat-inactivated fetal bovine serum, 1% penicillin–streptomycin (10 U/mL penicillin, 100 μg/mL streptomycin), and 20 ng/mL recombinant mouse M-CSF (PeproTech, #315-02) at 37 °C in a humidified 5% CO_2_ incubator. After 4 days, floating cells and debris were discarded; the adherent fraction was replenished with fresh medium containing 20 ng/mL M-CSF and maintained for macrophage differentiation. Over a period of 7 to 10 days, the medium was refreshed every 2–3 days until the cells were ready for use in the designated experiments. Additionally, the mouse aortic vascular smooth muscle cell line (MOVAS, ATCC, CRL-2797) and the human umbilical vein endothelial cell line (HUVEC, ATCC, CRL-1730) were maintained in high-glucose DMEM (4.5 g/L) containing 10% (v/v) heat-inactivated FBS, 10 U/mL penicillin, and 100 μg/mL streptomycin.

### Animals

Seven-week-old male Apolipoprotein E-deficient mice (*ApoE* ^–/–^ with C57BL/6J background), and C57BL/6J mice were purchased from fukang Hua biotechnology Co. Ltd. (Beijing, China). To establish atherosclerotic plaque-bearing mice, the *ApoE* ^–/–^ mice were fed with a high-fat diet (HFD, containing 20% fat,1.25% cholesterol, 45% carbohydrate, and 23% protein, Research Diets Inc., D12108C). To induce accelerated plaque rupture, 12 week-old *ApoE* ^–/–^ mice were received an HFD for 4 weeks, and then implanted with subcutaneously with osmotic minipumps (Alzet model 2004, 28 day delivery; Durect Corporation, USA) containing angiotensin II (1000 ng/kg/min, A9525; Sigma, MO, USA), while continuing the HFD for another 4 weeks. These angiotensin-infused plaque-bearing *ApoE* ^–/–^ mice were subsequently used for anti-atheroclerosis studies, mechanistic investigations, and biocompatibility evaluation. All animal procedures were conducted under protocols reviewed and approved by the institutional animal care and use committees of Sichuan University’s State Key Laboratory of Biotherapy, approval number 20230928223, and of Brigham and Women’s Hospital, Harvard Medical School, and Academia Sinica. This study did not involve human participants or human-derived samples.

### Preparation of FW-LP@siIRF5 Nanoparticles

The empty cationic liposomes were prepared using a thin-film dispersion technique.^35^ In brief, all organic components (molar ratios in Table S6) were dissolved in a 1:1 (v/v) chloroform/ethanol mixture to obtain the liposome precursors. The organic solvents were evaporated to form a uniform film with a rotary evaporator under vacuum at 37 °C for 2 h. Subsequently, The lipid film was rehydrated with 2 mL of RNase-free water at 60 °C for 30 min, yielding a suspension whose DOTAP concentration was adjusted to 1.5 mg mL^–1^. The suspension was then sonicated at 100 W (on/off cycle: 3/3 s) for 3 min using an ultrasound probe and filtered through a 0.22 μm sterilized filter for subsequent studies.

For siRNA loading, the positively charged FW-LP was mixed with siRNA solution in RNase-free water at an LPs/siRNA weight ratio of 5:1. Then, the complexes were incubated for 30 min at room temperature to bind siRNA onto the FW-LP via electrostatic interaction. The prepared samples were denoted as FW-LP@siIRF5. The scrambled siRNA (siScr)-loaded FW-LPs, abbreviated as FW-LPs@siScr, were used as control siRNA-loaded LPs to investigate the functions of siIRF5. Similarly, the PEG-LP@siIRF5 was obtained using the same strategy and served as a control to assess the effects of cell-penetrating peptide WRK and target ligand FA. To calculate the encapsulation efficiency (EE) of siIRF5 or tracing the distribution of LPs@siIRF5 in vitro and in vivo, the PEG-LP@Cy5-siIRF5 or FW-LP@Cy5-siIRF5 was prepared and stored in a dark environment to protect from light.

### Cellular Uptake Study

For cellular uptake studies, BMDMs were seeded onto glass-bottom dishes (NEST Biotechnology, Wuxi, China) at 1 × 10^5^ cells per dish and allowed to adhere overnight. After attachment, the cells were cultured for 12 h in fresh medium supplemented LPS (50 ng/mL) and IFN-γ (50 ng/mL). Subsequently, the cells were subjected to free Cy5-siIRF5, PEG-LP@Cy5-siIRF5, or FW-LP@Cy5-siIRF5 at an equivalent concentration of 50 nM Cy5-siIRF5 for an additional 4 h. Following incubation, the cells were rinsed three times with PBS and stained by Hoechst 33342 (Thermo Fisher Scientific, catalog no. H1399) for 15 min. After staining, the cells were rinsed again (three cycles) and then imaged using a confocal fluorescence scanning system with 633 nm laser excitation (Carl Zeiss LSM880). For flow cytometry experiments, cells underwent similar treatment, were subsequently harvested, suspended in PBS, and analyzed using a fluorescence-activated cell sorting system at an excitation wavelength of 633 nm (FACS, AccuriC6, BD Biosciences). All cytometric analyses were performed in FlowJo software and GraphPad Prism 8.

### Evaluation of Lysosomal Escape of FW-LP@siIRF5

BMDMs were plated at 1 × 10^5^ cells per glass-bottom dish (NEST Biotechnology, Wuxi, China) and allowed to adhere overnight. Lysosomes were subsequently visualized with LysoTracker Green (Thermo Fisher Scientific, L7526). After a 1 h incubation, the cells were rinsed three times with PBS and subsequently treated with FW-LP@siIRF5 at a concentration of 50 nM Cy5-siIRF5 for either 1 or 4 h. Following treatment, the medium was aspirated, and the cells were gently washed three times with PBS before staining with Hoechst 33342 to label the cell nuclei. Subsequently, the cell images were were examined and captured using a confocal laser scanning microscope (Carl Zeiss LSM880), with excitation set at 488 nm for LysoTracker Green and 633 nm for Cy5-siRNA.

### Gene Silencing Efficiency of FW-LPs@siIRF5In Vitro

The extent of gene knockdown of different siRNA formulations was quantified by measuring mRNA levels using quantitative real-time PCR (qRT-PCR) and Western blotting for protein expression. For the qRT-PCR experiments, BMDMs were seeded in a 6-well plate at a density of 3 × 10^5^ cells per well and incubated for 24 h. The cells were then pretreated with LPS (50 ng/mL) and IFN-γ (50 ng/mL) for 12 h, followed by incubattion with either fresh medium (control group) or different formulations, including free siIRF5, PEG-LP@siIRF5, FW-LP@siIRF5, or FW-LP@siScr, all at an equivalent concentration of 50 nM siRNA, for 48 h. Following treatment, the medium was aspirated and total RNA isolated with TRIzol reagent (Takara) per the vendor’s protocol. RNA was subsequently converted to cDNA with the PrimeScript RT kit (Takara), and IRF5 transcript abundance was quantified by qRT–PCR on a Bio-Rad CFX96 instrument with gene-specific primers (Table S7). GAPDH was employed as an internal reference for data normalization, and relative mRNA abundance was determined via the (2^–ΔΔCt^) algorithm.

For Western blot analysis, cells were treated for 12 h with 50 ng mL^–1^ LPS plus 50 ng mL^–1^ IFN-γ., followed by incubation with fresh medium or different formulations for 48 h. The cells were then detached, lysed in a Thermo Fisher Scientific FS30D bath sonicator. The supernatant was collected after centrifugation (12,000*g*, 15 min, 4 °C) and its total protein content was quantified with the BCA Protein Assay Kit (Beyotime, China). Protein extracts were resolved by 10% SDS-PAGE and subsequently electro-transferred onto a polyvinylidene difluoride membrane (PVDF). After 1 h of blocking at room temperature, the membranes were probed with rabbit polyclonal anti-IRF5 (Abcam, ab21689; 1 μg/mL) overnight at 4 °C, then incubated for 2 h at room temperature with HRP-conjugated goat antirabbit IgG (Abcam, ab205718; 1:10,000). GAPDH served as the loading control (Abcam, ab245355; 1:5000). Western blot chemiluminescence was generated with an ECL kit (Millipore) and captured on a ChemiDoc XRS imager (Bio-Rad); band densities were analyzed in Image-Pro Plus 6.0.

### Determination of Macrophage Phenotype In Vitro

The macrophage phenotype was assessed using both flow cytometry (for quantitative analysis) and immunofluorescence staining (for qualitative analysis). For flow cytometry, mouse bone-marrow-derived macrophages (2 × 10^5^ cells per well) were plated in 24-well dishes, allowed to adhere overnight, and subsequently exposed to LPS (50 ng/mL) plus IFN-γ (50 ng/mL) for 12 h, followed by treatment with either fresh medium (model of M1-like macrophages) or various formulations, including free siIRF5, PEG-LP@siIRF5, FW-LP@siIRF5, or FW-LP@siScr, all at an equivalent concentration of 50 nM siRNA, for an additional 24 h. Cells were detached with 0.25% trypsin/0.03% EDTA, spun down (1000 rpm, 3 min), rinsed twice in PBS, and resuspended in 100 μL of FACS buffer (D-PBS plus 2% FBS, 0.5% BSA and 0.05% NaN_3_). Cell suspensions were first incubated with FC Block (BD Biosciences, 553141) for 10 min, then labeled with BV421-conjugated antimouse F4/80 (BD Biosciences, 565411, 2.5 μg/mL) at 4 °C for 45 min. After that, the cell suspensions were washed with FACS buffer, fixed with Fix/Perm buffer (BD Biosciences), and incubated with FITC antimouse CD206 (MMR) antibody (BioLegend, 141706, 2.5 μg/mL) and Alexa Fluor 647 antimouse iNOS (Cell Signaling Technology, #48866, 1.5 μg/mL) at 4 °C for another 30 min. Finally, the CD206^+^ and iNOS^+^ expression levels in macrophages were quantified using flow cytometry (BD Biosciences San Jose, CA, USA), and flowJo software was used to analyze the results.

For immunofluorescence, the cells were plated at 2 × 10^5^ per glass-bottom dish, primed with LPS (50 ng mL^–1^) plus IFN-γ (50 ng mL^–1^) for 12 h, and then exposed to fresh medium or test formulations for a further 24 h. After fixation in 4% paraformaldehyde and permeabilisation with 0.1% Triton X-100, nonspecific binding was blocked with 1% BSA (40 min). Samples were incubated overnight at 4 °C with rabbit anti-CD206 (Abcam ab64693, 1:1000) and anti-iNOS (Abcam ab3523, 1:20), followed by Alexa-Fluor-594-conjugated goat antirabbit IgG (ab150088, 1:1000) and Hoechst 33342 (1 μg mL^–1^). Images were acquired on a Zeiss LSM880 confocal microscope and analyzed with Image-Pro Plus 6.0. Cytokine levels (TNF-α, IL-6, IL-1β, IL-10) in culture supernatants were quantified by commercial ELISA kits according to the supplier’s protocols.

### In Vivo Pharmacokinetics Evaluation

To evaluate in vivo pharmacokinetics, *ApoE* ^–/–^ mice with atherosclerotic plaques were randomly assigned to four groups (*n* = 3 per group). Each group was administered intravenously with either free Cy5-siIRF5 or different Cy5-labeled siIRF5 formulations, including PEG-LP@Cy5-siIRF5, WRK-LP@Cy5-siIRF5, FA-LP@Cy5-siIRF5, and FW-LP@Cy5-siIRF5 at a dose of 20 μg of Cy5-siIRF5 per mouse. Blood samples (approximately 20 μL) were collected from the orbital vein of each mouse using a heparin-coated pipet tip at 0, 10, 20, 40 min, and 1, 2, 4, 8, 12 h post injection. The fluorescence intensity of Cy5-siIRF5 in the blood samples was quantified with a microplate reader (BioTek, USA) using 625 nm excitation and 670 nm emission. Blood circulation time was calculated by measuring the percentage of Cy5-siIRF5 still present in the bloodstream at different time points, using the initial measurement as the baseline.

### In Vivo Biodistribution and Plaque-Targeting Capability Assessment

To assess biodistribution, atherosclerotic plaque-bearing *ApoE* ^–/–^ mice were received intravenous administration of saline, free Cy5-siIRF5, or various Cy5-labeled siIRF5 formulations, including PEG-LP@Cy5-siIRF5, WRK-LP@Cy5-siIRF5, FA-LP@Cy5-siIRF5, and FW-LP@Cy5-siIRF5, at a dose of 20 μg of Cy5-siIRF5 per mouse. The saline-injected mice served as the control group. Twelve h postinjection, the aortas and other main organs (kidneys, lungs, spleen, and liver) were collected and imaged using an ex vivo near-infrared fluorescence (NIRF) imaging system (IVIS Lumina, PerkinElmer) at excitation and emission wavelengths of 649 and 670 nm, respecitvley. The imaging data were quantified using ImageJ software.

For assessing plaque-targeting capability, ice acetone-fixed aortic root sections were harvested from the treated mice and incubated overnight at 4 °C with an anti-CD68 antibody (Abcam, ab283654, rabbit, 2 μg/mL). The sections were then stained with Alexa Fluor 488-conjugated goat antirabbit IgG secondary antibody (Abcam, ab150077, 2 μg/mL) and DAPI. Imaging was performed using a confocal laser scanning microscope (Carl Zeiss LSM880), and image quantification was conducted using Image-Pro Plus 6.0 software.

### Flow Cytometry Analysis of Aortic and Blood Cells

Atherosclerotic plaque-bearing *ApoE* ^–/–^ mice were intravenously injected with free Cy5-siIRF5 and various Cy5-labeled siIRF5 formulations at a dose of 20 μg of Cy5-siIRF5 per mouse. Twelve h postinjection, aortic cells from the entire aorta and the blood cells were extracted from the mice, following previously described protocols.^6^ The extracted aortic cellular samples were subjected to flow cytometry measurement, using nonimmune cells and lesional macrophage immunophenotyping panels using the following antibodies (listed in Table S3): PerCP-Cy5.5-anti-CD45 (clone 30-F11; 550994, BD Biosciences), PE-Cy7-anti-CD11b (clone M1/70, 552850; BD Biosciences), and BV421-anti-F4/80 (clone BM8; 565411, BD Biosciences). Lesional macrophages from digested aortas were identified as CD45^+^CD11b^hi^F4/80^hi^ cells, while nonimmune cells were identified as CD45^–^ cells.

Similarly, myeloid and lymphoid cells in the blood were identified through flow cytometry using the following antibodies (listed in Table S4): PerCP-Cy5.5-anti-CD45 (clone 30-F11; 550994, BD Biosciences), FITC-anti-CD90.2 (clone 53–2.1; 561973, BD Biosciences), FITC-anti-CD45R (clone RA3–6B2; 553088, BD Biosciences), FITC-anti-CD49b (clone DX5; 553857, BD Biosciences), FITC-anti-NK1.1 (clone PK136; 553164, BD Biosciences), FITC-anti-Ter119 (clone TER119; 116206, BioLegend), FITC-anti-Ly6G (clone 1A8; 127606, BioLegend), PE-Cy7-anti-CD11b (clone M1/70, 552850; BD Biosciences), and BV605-Ly-6C (clone AL-21, 563011; BD Biosciences). Ly-6C^hi^ monocytes were identified as CD45^+^CD11b^hi^Lin^–/low^Ly-6C^hi^ cells (with Lin^+^ defined as CD90.2^+^CD45R^+^CD49b^+^NK1.1^+^Ly-6G^+^Ter119^+^). Neutrophils were identified as CD45^+^CD11b^hi^Lin^hi^ cells. Ly-6C^low^ monocytes were identified as CD45^+^CD11b^hi^Lin^–/low^Ly-6C^low^ cells. Lymphocytes were gated as CD45^+^CD11b^low^ Lin^+^ cells events. Acquisition was performed on a BD LSRFortessa cytometer with FACSDiva, and downstream analysis was carried out in FlowJo.

### In Vivo Therapeutic Evaluation of siRNA Formulations in Atherosclerotic *ApoE* ^–/–^ Mice

After feeding HFD for 8 weeks, atherosclerotic *ApoE* ^–/–^ mice with established plaques were randomly assigned to four groups (*n* = 8). The mice were received intravenously with saline or different siRNA formulations (PEG-LP@siIRF5, FW-LP@siIRF5, and FW-PEG-LP@siScr) at an equivalent dosage of 20 μg siIRF5 or siScr per mouse via tail vein twice weekly for a further 8 weeks, while being maintained on the HFD. The unmodified PEG-LP@siIRF5 served as a negative control to assess the enhanced therapeutic effects of the cell-penetrating peptide WRK and the targeting ligand FA. FW-PEG-LP@siScr, loaded with scrambled siRNA (siScr), was used to confiem the antiatherosclerotic therapeutic effect of siIRF5.

One week after the final administration, *ApoE* ^–/–^ mice received isoflurane anesthesia and were sacrificed; blood was drawn by left-ventricular puncture. Cold heparinized PBS (1000 U/mL) was then slowly infused through the same route, maintaining plaque integrity within the vessel wall. Following perfusion, both entire aortas and aortic roots were harvested from the treated *ApoE* ^–/–^ mice and subjected to various pathological procedures to assess the total lesion areas and the stability of atherosclerotic plaques. Briefly, en-face aortas and frozen sections of aortic roots were stained with Oil Red O (ORO) to evaluate the level of atherosclerotic lesions. Additionally, to quantify necrotic core and collagen content, serial aortic-root sections were stained with H&E or Masson’s trichrome; each lesion was digitized on a Pannoramic MIDI scanner and the respective areas were normalized to total wall area in Image-Pro Plus 6.0.

### In Vivo IRF5 Gene Silencing and Lesional Macrophage Phenotype Reprogramming

To evaluate the in vivo efficiency of IRF5 gene silencing and its impact on reprograming the phenotype of lesional macrophages, atherosclerotic plaque-bearing *ApoE* ^–/–^ mice were received twice-weekly intravenous injections over an 8 week period with saline or various siRNA nanomedicines (PEG-LP@siIRF5, FW-LP@siIRF5 or FW-LP@siScr) at an equivalent dosage of 20 μg siIRF5 or siScr per mouse. One week after the final administration, the aortas were harvested from the treated *ApoE* ^–/–^ mice. The tissue was enzymatically dissociated using a combination of collagenase I (450 U/mL, C0130, Sigma-Aldrich), collagenase XI (125 U/mL, C7657, Sigma-Aldrich), hyaluronidase (60 U/mL, H3506, Sigma-Aldrich), and DNase I (60 U/mL, D4527, Sigma-Aldrich) in Hank’s balanced salt solution at 37 °C for 1 h. The resulting cell suspension was passed through a 70 μm filter and rinsed twice with Dulbecco’s PBS to isolate single cells. Afterward, cells were rinsed in FACS buffer—DPBS containing 2% FBS, 0.5% BSA and 0.05% NaN_3_—then incubated with FC Block (553141, BD Biosciences) before a 45 min, 4 °C staining step with the indicated antibodies (listed in Table S5): PerCP-Cy5.5-anti-CD45 (clone 30-F11; 550994, BD Biosciences), PE-Cy7-anti-CD11b (clone M1/70, 552850; BD Biosciences), BV421-anti-F4/80 (clone BM8; 565411, BD Biosciences), PE-anti-IRF5 (158604; BioLegend), FITC-anti-CD206 (MMR) (141704; BioLegend), and Alexa Fluor 647-antimouse iNOS (#48866; CST). IRF5-expressing lesion-resident macrophages were gated as CD45^+^CD11b^high^F4/80^high^IRF5^+^; M2-like macrophages as CD45^+^CD11b^high^F4/80^high^CD206^+^; and M1-like macrophages as CD45^+^CD11b^high^F4/80^high^iNOS^+^. Acquisition was performed on a BD LSRFortessa cytometer with FACSDIVA (Becton Dickinson), followed by analysis in FlowJo.

### Evaluation of Adverse Immune Effects and Toxicity of FW-LP and FW-LP@siIRF5

To evaluate the in vivo adverse immune effects and toxicity of empty FW-LP or FW-LP@siIRF5, seven-week-old male C57BL/6J mice were randomly assigned to three experimental groups (*n* = 6 per group). The mice were intravenously injected with saline, empty FW-LP, or FW-LP@siIRF5 every 2 days for 14 days. During this 14 day period, mice were weighed at 48 h intervals. Forty-8 h after the last treatment, animals were sacrificed; heart, liver, spleen, lung and kidney specimens were taken for histopathology. Whole-blood samples were run on a Sysmex KX-21 analyzer for hematologic indices, and serum was processed on a Roche Cobas C501 to quantify hepatic (ALT, AST) and renal (BUN, CRE) markers. To evaluate possible immune reactions, residual serum was tested for the pro-inflammatory markers TNF-α, IL-1β and IL-6 with the corresponding ELISA kits. In addition, the safety profile of FW-LP@siIRF5 was investigated in atherosclerotic plaque-bearing *ApoE* ^–/–^ mice after 8 weeks of treatment with saline or different siRNA formulations. Serum levels of total cholesterol, triglycerides, fasting glucose, and LDL in atherosclerotic *ApoE* ^–/–^ mice were quantified with a fully automated Roche Cobas C501 analyzer (Roche, Switzerland) following the respective interventions.

### Additional Methods

Comprehensive synthetic protocols and experimental details are available in the Supporting Information, including Synthesis of WRK-PEG_2k_-Chol and FA-PEG_2k_-Chol; Physicochemical characterization and stability of FW-LP@siIRF5 nanoparticles; siIRF5 release assay; in vitro cytotoxicity assay; cell viability assay in plaque-derived primary VSMCs and mouse aortic endothelial cells (MAEC); uptake mechanism assay; quantitative real-time PCR assay analysis of macrophage repolarization-associated gene expression; acute MRSA-induced infection model; and in vitro cytotoxicity assay.

### Quantification and Statistical Analysis

Data distribution was initially screened with the D’Agostino-Pearson or Shapiro–Wilk test. To compare two groups under the assumption of normality, we applied a two-tailed Student’s *t* test, while one-way analysis of variance (ANOVA) with Tukey posthoc, Dunnett’s T3, or Games-Howell posthoc tests was used for analyzing differences between more than two groups. When data deviated from normality, pairwise comparisons were performed with the Mann–Whitney U test, whereas multigroup comparisons relied on the Kruskal–Wallis test. Results were deemed noteworthy at *P* < 0.05; increasing levels of evidence were flagged as *P < 0.05, **P < 0.01 and ***P < 0.001. Values are reported as mean ± SD, and calculations were carried out in GraphPad Prism 8.0 (GraphPad, USA).

## Supplementary Material



## Data Availability

The data supporting the findings in this study are available within the paper and its. All data generated in this study are available from the lead contact on reasonable request.

## Notes

Detailed experimental procedures; synthesis and physicochemical characterization of lipid–peptide conjugates and nanoparticles; dynamic light scattering size analysis, encapsulation efficiency, serum stability, and release profiles; in vitro cellular uptake, viability assays, macrophage polarization analyses, and pathway-related gene expression studies; ex vivo biodistribution imaging; additional histological and plaque stability assessments; flow cytometry gating strategies; extended in vivo therapeutic evaluations in angiotensin-infused plaque-bearing mice; biosafety and immune compatibility assessments; supplementary tables summarizing nanoparticle characteristics, primer sequences, antibody information, and formulation compositions (PDF)
