# Supplementary material for: Reprogramming Lesional Macrophage Homeostasis via Interferon Regulatory Factor 5 Targeted siRNA Nanoimmunotherapy for Atherosclerosis
Source: ACS Nano. 2026 Mar 4;20(10):8350–71. doi: 10.1021/acsnano.5c18044 (PMC13001080; doi:10.1021/acsnano.5c18044)
Supplement: Supplementary file 1 [file nn5c18044_si_001.pdf]

Supplementary Information

Reprogramming Lesional Macrophage Homeostasis *via* Interferon Regulatory Factor 5 Targeted siRNA Nanoimmunotherapy for Atherosclerosis

Zhongshan He[1,2,# *], Yaoyao Luo[3#], Shuping Yang[1], Haixing Shi[1], Ya-Chih Huang[4], Zhuoming Zhou[5], Shengbin Liu[1],Wanqin Zeng[1], Wei-Chieh Liu[4], Yongjiang Li[5], Yuting Chen[1], Duotian Qin[5], Xing Duan[1], Xi He[1], Wei Chen[4*], Xiangrong Song[1*]

[1]Department of Clinical Pharmacy, Frontiers Science Center for Disease-related Molecular Network, State Key Laboratory of Biotherapy and Cancer Center, West China Hospital, Sichuan University, Tianfu Jincheng Laboratory, Chengdu, China;

[2]Departments of Molecular Physiology and Biological Physics & Biomedical Engineering, Cardiovascular Research Center, Virginia University, Charlottesville, VA 22903, USA;

[3]Laboratory of Cardiac Structure and Function ，Institute of Cardiovascular Diseases ，West China Hospital, Sichuan University, Chengdu, China;

[4]Genomics Research Center, Academia Sinica, Taipei 115, Taiwan;

[5]Center for Nanomedicine and Department of Anesthesiology, Brigham and Women's Hospital, Harvard Medical School, Boston, MA 02115, USA

[*]These authors contributed equally

[#]Lead contact

[*]Correspondence: songxr@scu.edu.cn (X.S.); wchen123@as.edu.tw (W.C.); zhongshan_he@163.com (Z.H.)

Table of Contents

1. Supplementary Experimental Procedures ………………………………………………………... 4

2. Supplementary Figure..7

Supplementary Figure S1 Synthesis and characterization of WRK-PEG2k-Chol 7

Supplementary Figure S2 Synthesis and characterization of FA-PEG2k-Chol 8

Supplementary Figure S3 Time-dependent dynamic light scattering (DLS) size and siRNA encapsulation efficiency 9

Supplementary Figure S4 Protection of siRNA against serum nucleases 9

Supplementary Figure S5 Release profiles of siIRF5 from FW-LP@siIRF5 under physiological and plaque-mimicking conditions 10

Supplementary Figure S6 Celluar uptake mechanism of FW-LP@siIRF5 10

Supplementary Figure S7 *In vitro* cell viability assay..11

Supplementary Figure S8 Investigation of the M1/M2 marker expression levels in BMDMs 12

Supplementary Figure S9 Expression of macrophage repolarization-related pathway genes following FW-LP@siIRF5 treatment..12

Supplementary Figure S10 Biodistribution study using ex vivo near-infrared fluorescence imaging.....14

Supplementary Figure S11 Enhanced accumulation of FW-LP@Cy5-siIRF5 within lesional macrophages 15

Supplementary Figure S12 ORO staining assay demonstrating the superior anti-atherosclerotic efficacy of FW-LP@siIRF5 treatment 16

Supplementary Figure S13 FW-LP@siIRF5 therapy reduces plaque burden and improves features of plaque stability..17

Supplementary Figure S14 Flow cytometry gating strategy for IRF5 expression and lesional macrophage phenotype 18

Supplementary Figure S15 FW-LP@siIRF5 therapy promotes anti-inflammatory macrophage polarization and reduces inflammatory signaling..19

Supplementary Figure S16 FW-LP@siIRF5 therapy reduces IRF5 expression in lesional macrophages of plaque-bearing, angiotensin-infused *ApoE*$^{-/-}$ mice..21

Supplementary Figure S17 FW-LP@siIRF5 therapy promotes anti-inflammatory macrophage polarization and reduces inflammatory signaling in plaque-bearing, angiotensin-infused *ApoE*$^{-/-}$ mice .22

Supplementary Figure S18 Time-course body weight of atherosclerotic *ApoE*$^{-/-}$ mice during various treatments..23

Supplementary Figure S19 Biosafety Assessment in angiotensin infusion-induced atherosclerotic mice following various treatments 24

Supplementary Figure S20 *In vivo* evaluation of adverse immune effects and biocompatibility of empty FW-LP or FW-LP@siIRF5 26

Supplementary Figure S21 FW-LP@siIRF5 does not impair host inflammatory responses in an acute MRSA bacteremia model 27
3. Supplemental Tables 28
Table S1 Characteristics of LPs and LPs@siRNA nanoparticles measured by DLS........28
Table S2. Primer sequences used for the RT-qPCR analysis. 28
Table S3 Antibodies used for the flow cytometry analysis of lesional macrophages. 28
Table S4. Antibodies used for the flow cytometry analysis of blood cells. 28
Table S5 Antibodies used for the flow cytometry analysis of IRF5 expression and M2-like and M1-like macrophages expression in aortic lesional macrophages. 29
Table S6 Composition of LPs formulations. 29
Table S7 Primer sequences used for the RT-qPCR analysis 29

1. Supplementary Experimental Procedures

Synthesis of WRK-PEG2k-Chol and FA-PEG2k-Chol

For the synthesis of WRK-PEG_{2k}-Chol, 500 mg of Chol-PEG_{2K}-COOH, a total of 17.2 mg each of *N*-Hydroxysuccinimide (NHS), and of 1-(3-Dimethylaminopropyl)-3-ethylcarbodiimide hydrochloride (EDC•HCl) were dissolved in 2 mL of dimethylformamide (DMF) and stirred at room temperature for 2 h. Subsequently, WRK, which had been pre-dissolved in DMF, was added to the reaction mixture. The resulting solution was continuously stirred for an additional 72 h at room temperature. Subsequently, the DMF solvent and any remaining Chol-PEG_{2K}-COOH, NSH, and EDC•HCl were removed by filtration (MWCO = 2,000 Da). The resulting product, WRK-PEG_{2k}-Chol, was obtained and any residual moisture was eliminated through lyophilization. Finally, the product was stored in a vacuum-sealed container and protected from light.

Similarly, for the synthesis of FA-PEG_{2k}-Chol, folic acid (1.77 g), DMAP (0.2 mmol, 0.24 g, 0.2 eq) and EDC•HCl (4 mmol, 0.77 g, 2 eq) were dissolved in 10 mL of anhydrous DMF under nitrogen. The mixture was then stirred at ambient temperature for 5 h. Subsequently, Chol-PEG_{2k}-OH (2 mmol, 4.91 g, 1 eq) was transferred to the solution, and stirring was continued for an additional 72 h. Subsequently, the DMF solvent and any residual ingredients were removed by filtration (MWCO = 1,000 Da). The sample was lyophilized to eliminate residual moisture, which resulted in the obtainment of FA-PEG_{2k}-Chol, the final product.

Physicochemical characterization and stability of FW-LP@siIRF5 nanoparticles

Hydrodynamic diameter and surface charge of LPs@siIRF5 were determined with a Malvern Zetasizer Nano-ZS (ZEN3600). FW-LP@siIRF5 morphology was visualized by transmission electron microscopy (TEM, Hitachi HT7700, 100 kV).

The encapsulation efficiency (EE) of siIRF5 in PEG-LP@siIRF5 and FW-LP@IRF5 was defined as (loaded siIRF5/total siIRF5) × 100%. To calculate the EE of siIRF5, we measured the fluorescence intensity of the extracted Cy5-siIRF5 from PEG-LP@Cy5-siIRF5 or FW-LP@Cy5-siIRF5 by an Infinite M200 PRO spectrophotometer (TECAN) and the fluorescence intensity was compared to a free Cy5-siIRF5 (1 nM). To assess the storage stability of LP@Cy5-siIRF5 in physiological environments, the changes in average particle size and encapsulation efficiency were monitored over a 7-day period.

To evaluate how well the lipoplexes (LPs) shield siIRF5, equal volumes of naked siIRF5, PEG-LP@siIRF5, or FW-LP@siIRF5 were mixed with undiluted serum and kept at 37°C for 12 h. Afterward, the complexes were dissolved with chloroform, and the RNA was recovered into 0.5 M NaCl/0.1 % SDS. The intactness of the released siRNA was then compared to that of the untreated control by running both on 4 % ethidium-bromide–agarose E-Gels (Bio-Rad) and imaging under UV light.

siIRF5 release assay

FW-LP@siIRF5 dispersions were prepared in PBS containing 20% (v/v) fetal bovine serum. To mimic the atherosclerotic plaque microenvironment, the pH was adjusted to 6.3 by adding dilute HCl or NaOH, followed by the addition of H_2O_2 (final concentration 1 mM). Samples were incubated at 37°C under continuous agitation (100 rpm) in the dark. At predetermined time points, aliquots were collected and immediately subjected to ultrafiltration to separate the

released siIRF5. The amount of released siRNA was quantified using the Qubit RNA HS assay, and cumulative release–time profiles were generated. Throughout the assay, sterility, ionic strength, and light protection were carefully maintained to ensure accurate simulation of plaque-like conditions.

***In vitro* cytotoxicity assay**

For the cell viability test, atherosclerosis-associated cell line, including RAW264.7, MOVAS, and HUVECs cells, were plated into 96-well plates at 5×10^3 cells per well and maintained in 100 µL of growth medium for 24 h. Following this, the cells were treated with either fresh medium (control group) or different formulations, such as free siIRF5, PEG-LP@siIRF5, FW-LP@siIRF5, or FW-LP@siScr, all at an equivalent concentration of 50 nM siRNA for 48 h. After the treatment period, the medium was then replaced with fresh medium supplemented with MTT (5 mg mL^{-1}, 1 µL per 10 µL medium) and incubated for a further 4 h at 37 °C. Viability was quantified by reading the absorbance of the extracellular solution at 550 nm on a Bio-Rad 680 microplate reader (UK) .

Cell viability assay in plaque-derived primary VSMCs and MAECs cells

Primary lesional vascular smooth muscle cells (VSMCs) and mouse aortic endothelial cells (MAECs) were isolated from the aortas of plaque-bearing *ApoE*$^{-/-}$ mice using a combined enzymatic digestion protocol (collagenase type I/XI, hyaluronidase, and DNase I), followed by flow cytometric sorting with antibodies against CD45, α-SMA, and CD31. Plaque-derived primary VSMCs were identified as $CD45^-$α-SMA^+ cells; primary MAECs were identified as $CD45^-CD31^+$ cells. Isolated primary cells were cultured *ex vivo* and treated with various siIRF5 formulations for 24 h. Cell viability was then assessed using a CCK-8 assay, performed in parallel with viability assays in established cell lines. Consistent with the cell-line data, FW-LP@siIRF5 exhibited excellent biocompatibility in all plaque-derived primary cell types.

Uptake mechanism assay

BMDMs were seeded at 2×10^5 cells per well in 48-well plates and cultured for 24 h before the assay. Following overnight serum starvation (2% FBS), cells were pretreated for 30 min at 37°C with freshly prepared endocytic inhibitors (all from Sigma-Aldrich, St. Louis, MO) dissolved in warm complete medium: chlorpromazine (10 µg mL^{-1}; clathrin-mediated endocytosis), methyl-β-cyclodextrin (MβCD; 5 mM; caveolae/lipid-raft–mediated endocytosis), or amiloride (50 µM; macropinocytosis). Vehicle-control wells received 0.1% DMSO, matching the highest solvent content among inhibitor conditions.

After pretreatment, the medium was replaced with inhibitor-containing medium supplemented with FW-LP@Cy5-siIRF5 (50 nM siRNA equivalent; 25 µL per well), and cells were incubated for 4 h at 37°C in 5% CO_2. Subsequently, cells were washed three times with ice-cold PBS, detached with 0.05% trypsin–EDTA (2 min, 37°C), quenched with 10% FBS, pelleted (300 g, 5 min), and resuspended in 300 µL PBS for flow cytometry analysis. Cy5 fluorescence was measured on a BD FACSCanto II (excitation 635 nm, emission 670 nm), and 10,000 single-cell events were collected per sample. Uptake efficiency was calculated as the percentage of geometric mean fluorescence intensity (MFI) relative to the vehicle control, after subtraction of autofluorescence from untreated cells. Each inhibitor condition was assayed in

triplicate and independently repeated three times.

Quantitative real-time PCR assay analysis of macrophage repolarization -associated gene expression

Macrophage mRNA levels of inflammation- and resolution-related genes were measured by qRT–PCR after FW-LP@siIRF5 treatment. BMDMs were plated in six-well plates (3×10^5 cells/well), rested overnight, and pre-treated with LPS (50 ng mL^{-1}) and IFN-γ (50 ng mL^{-1}) for 12 h, followed by incubation with either fresh medium (M1-like control) or different siRNA formulations—including free siIRF5, FW-LP@siIRF5, and FW-LP@siScr—each at an equivalent siRNA concentration of 50 nM, for an additional 48 h. After treatment, the medium was removed and total RNA was isolated with TRIzol (Takara) following the supplier's protocol. cDNA was synthesized with the PrimeScript RT kit (Takara) and subjected to qRT-PCR on a Bio-Rad CFX96 with primers targeting PPAR and inflammatory genes (Table S2). Expression data were referenced to GAPDH.

Acute MRSA-induced infection model.

Eight-week-old male C57BL/6J mice received 0.1 mL of a methicillin-resistant *Staphylococcus aureus* (MRSA, ATCC 33591) suspension containing 5×10^7 CFU via tail-vein injection. 6 h after infection, mice were randomly assigned to treatment groups and administered 150 μL of either saline or FW-LP@siIRF5 (50 μg per mouse) via intravenous injection. Blood was drawn from the orbital sinus 6 h after treatment and centrifuged at 300 × g for 10 min to separate plasma. Levels of pro-inflammatory cytokines and chemokine (TNF-α, IL-6, IL-1β, Cxcl2) and the anti-inflammatory cytokine IL-10 were quantified using ELISA kits (Beijing Solarbio Science & Technology Co., Ltd). Mouse body weight was monitored throughout the experimental period.

2. Supplementary Figure

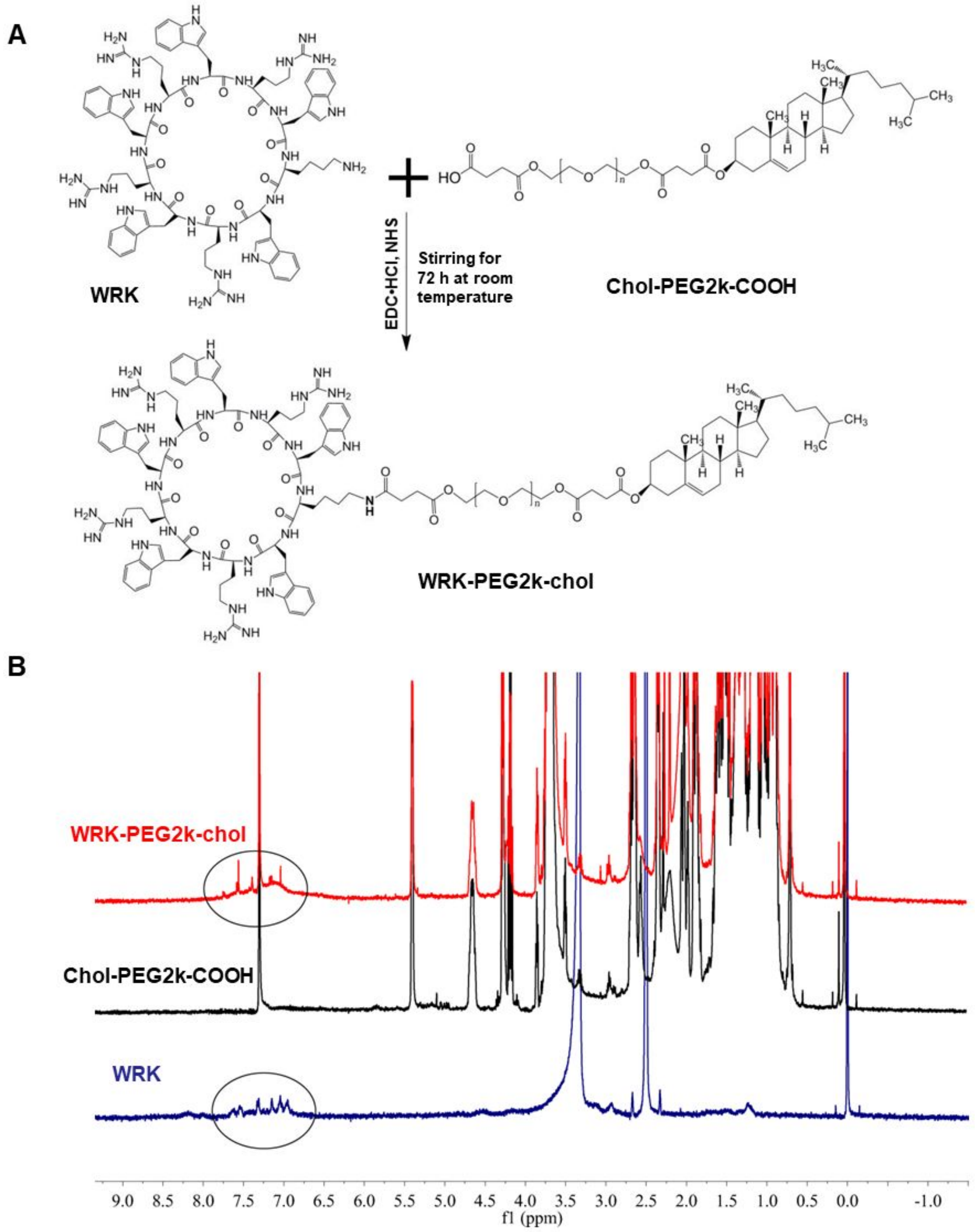

Figure S1. Synthesis and characterization of WRK-PEG2k-Chol. (A) Synthetic route of WRK-PEG_{2k}-Chol. The undecorated cyclic peptide WRWRWRWRWK (abbreviated as W5R4K or WRK) is composed of tryptophan (W), arginine (R), and lysine (K). (B) ^{1}H-NMR spectrometry of WRK-PEG_{2k}-Chol. NHS: *N*-Hydroxysuccinimide; EDC•HCl: 1-(3-Dimethylaminopropyl)-3-ethylcarbodiimide hydrochloride.

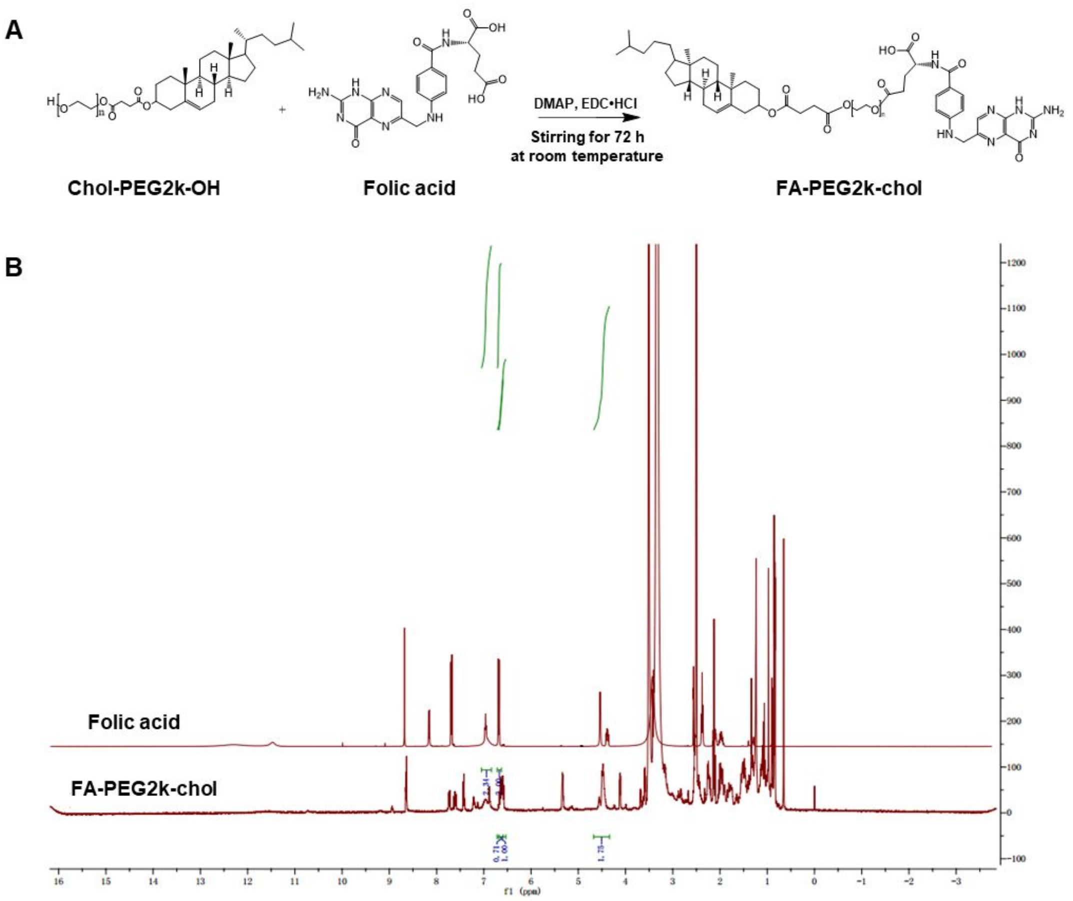

Figure S2. Synthesis and characterization of FA-PEG2k-Chol. (A) Synthetic route of FA-PEG$_{2k}$-Chol. (B) ^{1}H-NMR spectrometry of FA-PEG$_{2k}$-Chol. DMAP: dimethylaminopyridine; EDC•HCl: 1-(3-Dimethylaminopropyl)-3-ethylcarbodiimide hydrochloride.

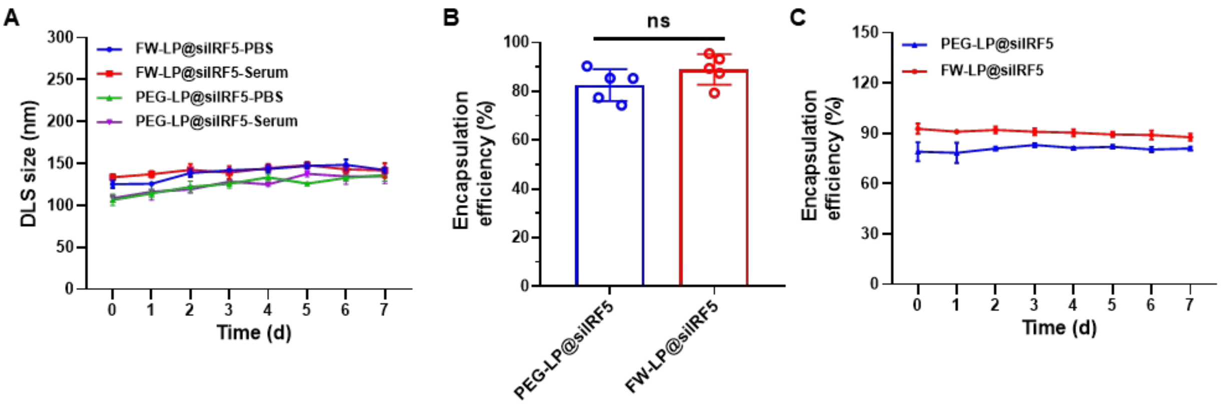

Figure S3. Time-dependent dynamic light scattering (DLS) size and siRNA encapsulation efficiency. (A) Change of DLS size over time. Stability of the PEG-LP@siIRF5 and FW-LP@siIRF5 over 7 day in PBS or medium containing 10% serum at 4°C (*n* = 3 biologically independent samples, mean ± S.D.). (B) Encapsulation efficiency and (C) time-dependent encapsulation efficiency of siIRF5 in PEG-LP@siIRF5 and FW-LP@siIRF5. PEG-LP and FW-LP were loaded with Cy5-labeled IRF5 siRNA (siIRF5) in PBS. The purified LPs were then extracted with dimethylsulfoxide, followed by quantification of the extracted Cy5 fluorescence. Encapsulation efficiency (EE) was calculated as the proportion of fluorescent siRNA incorporated into purified liposomes relative to the total amount initially used during nanoparticle preparation (*n* = 3 biologically independent samples, mean ± S.D.). Statistical significance was determined using Student's t-test, and ns denotes no significance.

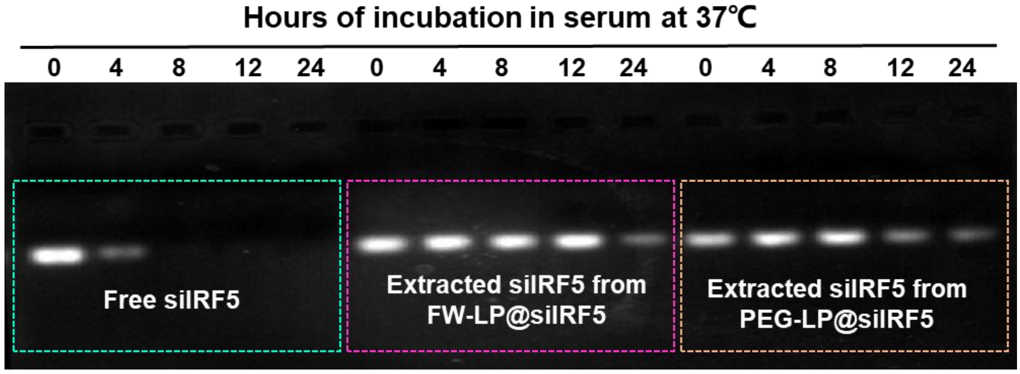

Figure S4. Protection of siRNA against serum nucleases. Free siIRF5, PEG-LP@siIRF5, and FW-LP@siIRF5 were incubated in 100% serum at a 1:1 volume ratio for 4, 8, 12, and 24 h at 37°C. Samples were then analyzed by agarose gel electrophoresis to assess siRNA stability and degradation over time.

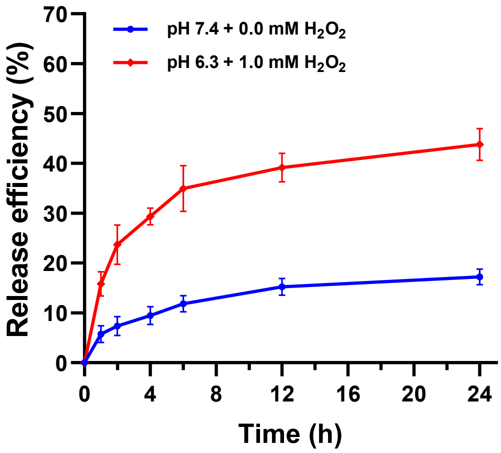

Figure S5. Release profiles of siIRF5 from FW-LP@siIRF5 under physiological and plaque-mimicking conditions. Time-dependent release of siIRF5 from FW-LP@siIRF5 was evaluated in PBS containing 20% fetal bovine serum under two environmental settings: (i) standard physiological conditions (pH 7.4, H_2O_2 = 0 mM) and (ii) atherosclerotic plaque–mimicking conditions (pH 6.3, H_2O_2 = 1.0 mM). The latter combination reflects the mildly acidic and oxidative microenvironment characteristic of inflamed atherosclerotic lesions. Data are presented as mean ± S.D. (n = 3 biologically independent samples).

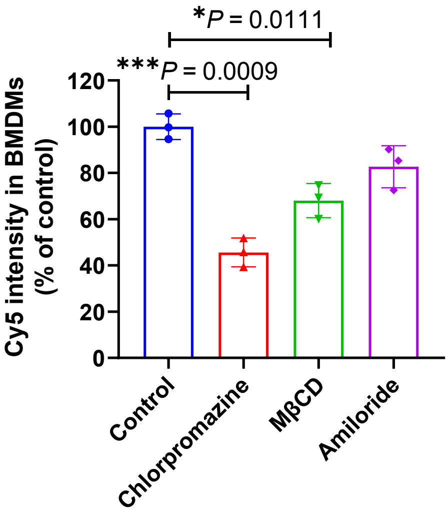

Figure S6. Celluar uptake mechanism of FW-LP@siIRF5. BMDMs were pretreated with chlorpromazine (10 µg mL^{-1}), methyl-β-cyclodextrin (MβCD, 5 mM), or amiloride (50 µM) before incubation with FW-LP@Cy5-siIRF5 (50 nM siRNA, 25 µL per well). After 4 h, uptake efficiency was quantified as the percentage of mean fluorescence intensity (MFI) relative to the vehicle control, following subtraction of untreated-cell autofluorescence. Each inhibitor condition was assessed in triplicate wells and independently repeated three times. Data are presented as mean ± S.D. (n = 3 biologically independent samples), and statistical significance was determined using one-way ANOVA with a Dunnett's T3 post hoc test. *P < 0.05, **P < 0.01, and ns denotes no significance.

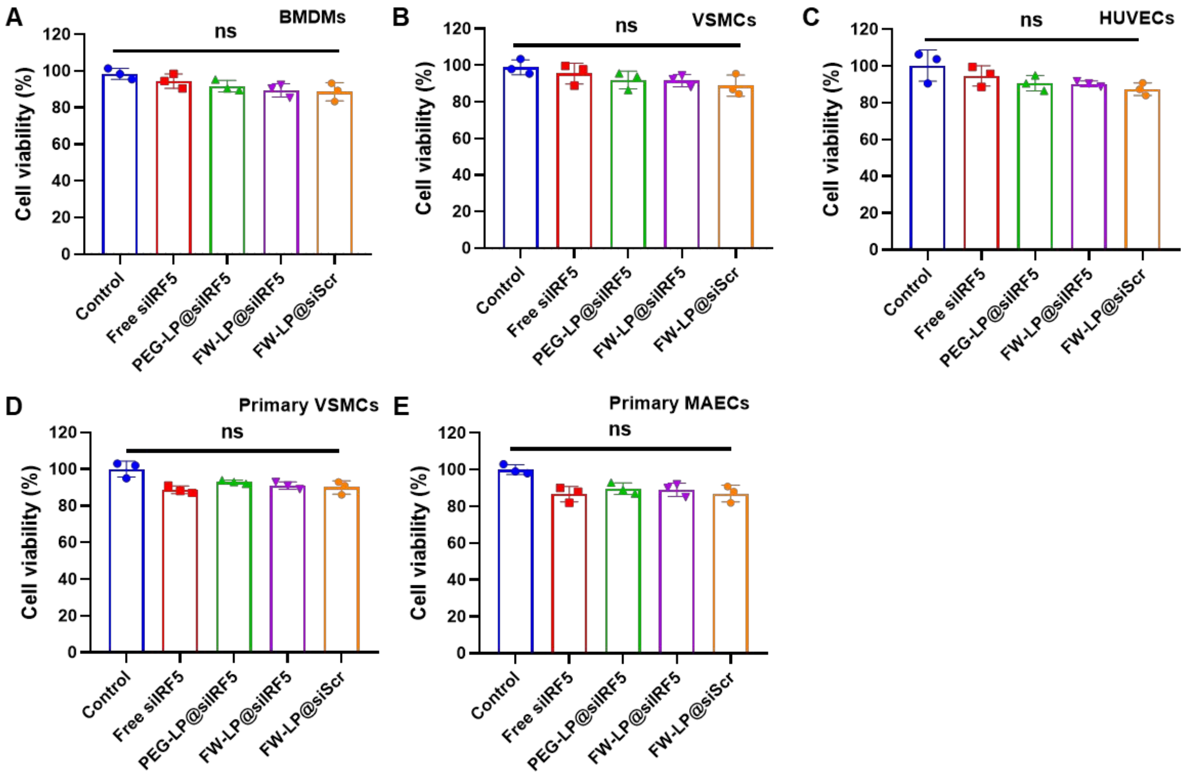

Figure S7. ***In vitro*** **cell viability assay.** (A) Bone marrow-derived macrophages (BMDMs), (B) vascular smooth muscle cells (VSMCs), (C) human umbilical vein endothelial cells (HUVECs), and (D-E) plaque-derived primary vascular smooth muscle cells (VSMCs) and mouse aortic endothelial cells (MAECs) were incubated with various siRNA formulations for 24 h. Primary lesional VSMCs and MAECs were isolated from the aortas of plaque-bearing *ApoE*$^{-/-}$ mice using a combined enzymatic digestion protocol (collagenase type I/XI, hyaluronidase, and DNase I), followed by flow cytometric sorting with antibodies against CD45, α-SMA, and CD31. Plaque-derived primary VSMCs were identified as CD45^{-}α-SMA^{+} cells; primary MAECs were identified as CD45^{-}CD31^{+} cells. Cells were incubated in 100 µL of fresh medium containing siIR5 at a concentration of 50 µg/mL for 24 h at 37°C. Cell viability was assessed using the MTT assay, and quantified by measuring the absorbance at 450 nm.Data are presented as mean ± S.D. (*n* = 3 biologically independent samples), and statistical significance was assessed using one-way ANOVA followed by Dunnett's T3 post hoc test. ns, not significant.

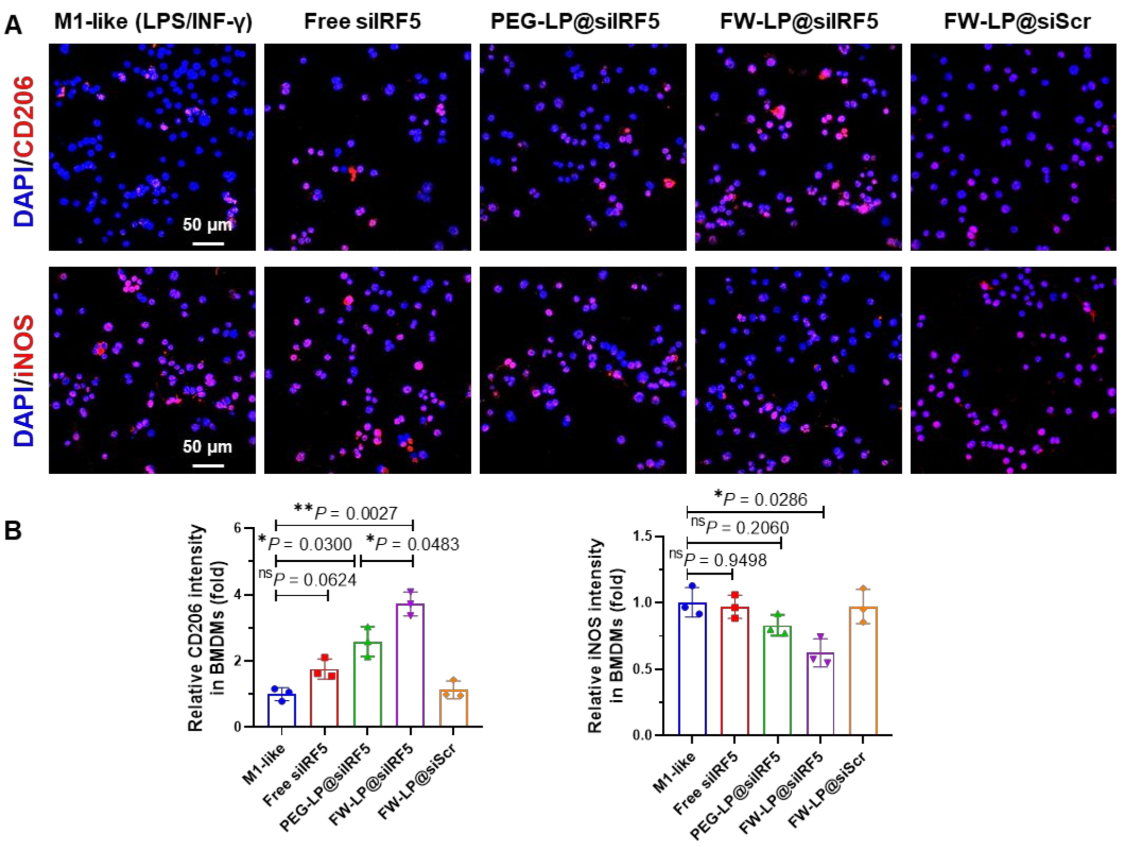

Figure S8. Investigation of the M1/M2 marker expression levels in BMDMs. (A) Representative confocal microscopy images showing expression of M2-like ($CD206^+$) and M1-like macrophages ($iNOS^+$) markers in LPS/INF-γ stimulated BMDMs treated with various siRNA formulations. Red: AF594-labeled CD206 or iNOS; Blue: DAPI-labeled cell nucleus. Scale bars, 50 μm. (B) Quantitative analysis of the relative fluorescence intensity of CD206 and iNOS in LPS/INF-γ-stimulated BMDMs treated with various siRNA formulations (n = 3 biologically independent samples, mean ± S.D.). Statistical significance was determined using one-way ANOVA with a Dunnett's T3 post hoc test. *P < 0.05, **P < 0.01, and ns denotes no significance.

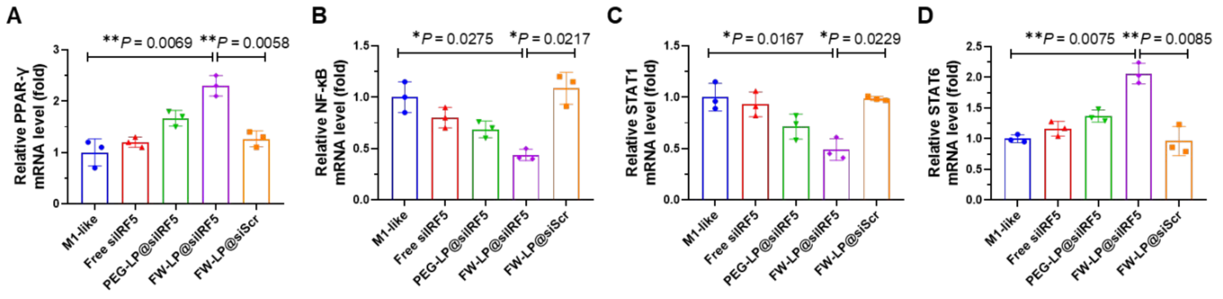

Figure S9. Expression of macrophage repolarization-related pathway genes following FW-LP@siIRF5 treatment. Relative mRNA expression of inflammatory-resolution genes (A) PPAR-γ and (D) STAT6, and (B–C) pro-inflammatory pathway genes NF-κB and STAT1, measured by RT-qPCR in LPS/IFN-γ–stimulated BMDMs treated with various siRNA

formulations. BMDMs were stimulated with LPS/IFN-γ for 12 h, followed by co-incubation with either fresh medium (M1-like control) or the indicated treatments for 48 h. Data were analyzed using one-way ANOVA with a Games-Howell post hoc test and shown as mean ± S.D. (n = 3 biologically independent samples). Statistical significance is indicated as $^*P < 0.05$, and $^{**}P < 0.01$.

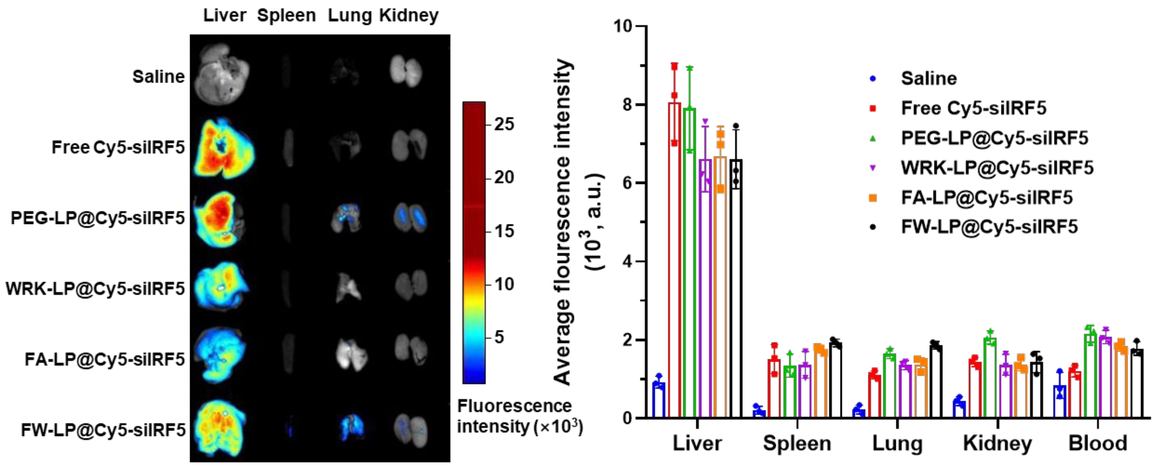

Figure S10. Biodistribution study using *ex vivo* near-infrared fluorescence imaging. *Ex vivo* near-infrared fluorescence images and corresponding quantitative analysis of fluorescence intensity in main organs (liver, spleen, lung, and kidney) collected 12 h after intravenous administration of saline, free Cy5-siIRF5, or various Cy5-labeled siIRF5 formulations (20 µg Cy5-siIRF5 per mouse) in atherosclerotic *ApoE*$^{-/-}$ mice. Data were shown as mean ± S.D. (n = 3 biologically independent mice).

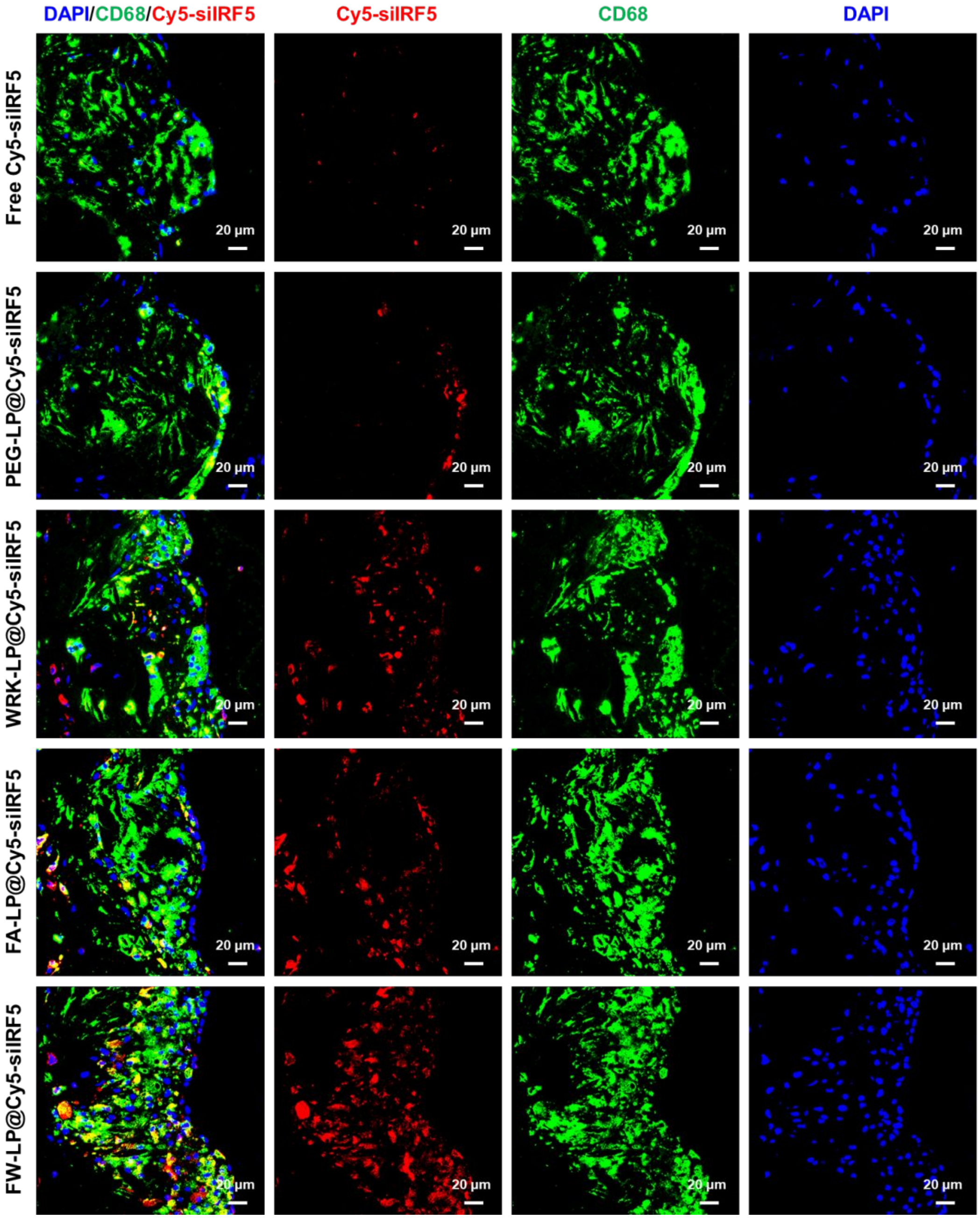

Figure S11. Enhanced accumulation of FW-LP@Cy5-siIRF5 within lesional macrophages. Confocal microscopy images illustrate the co-localization of FW-LP@Cy5-siIRF5 (red) with macrophages (green) in atherosclerotic aortic roots. The merged images are also displayed in Figure 4G. Red: Cy5-labeled LP@siIRF5 or free siIRF5; Green: macrophage marker stained with anti-CD68 antibody; Blue: cell nuclei stained with DAPI. Scale bars, 20 μm.

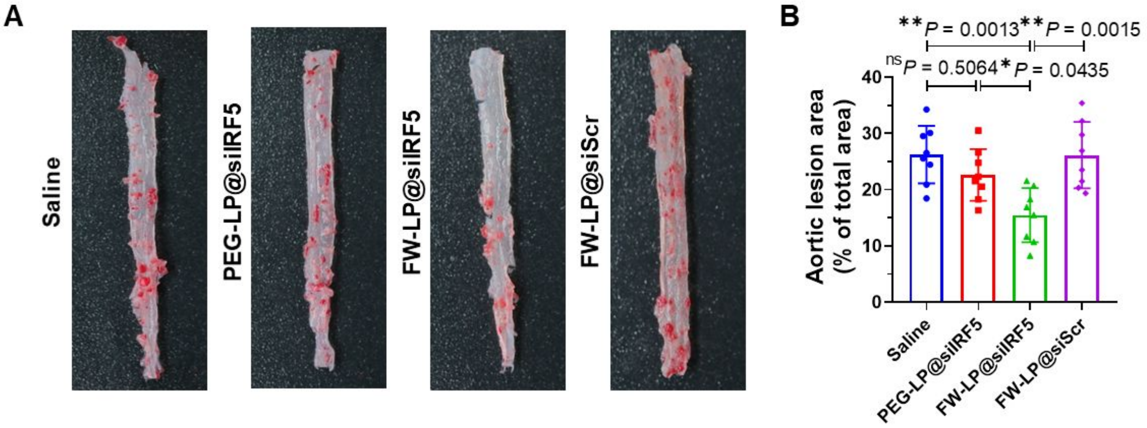

Figure S12. ORO staining assay demonstrating the superior anti-atherosclerotic efficacy of FW-LP@siIRF5 treatment. (A) Representative *en face* ORO-stained whole aortas from atherosclerotic mice after various siRNA treatments at equivalent dosage of 20 μg IRF5 siRNA per mouse. (B) Quantitative analysis of lesion area (ORO-positive area) in the entire aorta using ImageJ software (n = 8 biologically independent mice, mean ± S.D.). Group differences were analyzed by one-way ANOVA followed by Tukey's post hoc test. *$P < 0.05$, **$P < 0.01$, and ns denotes no significance.

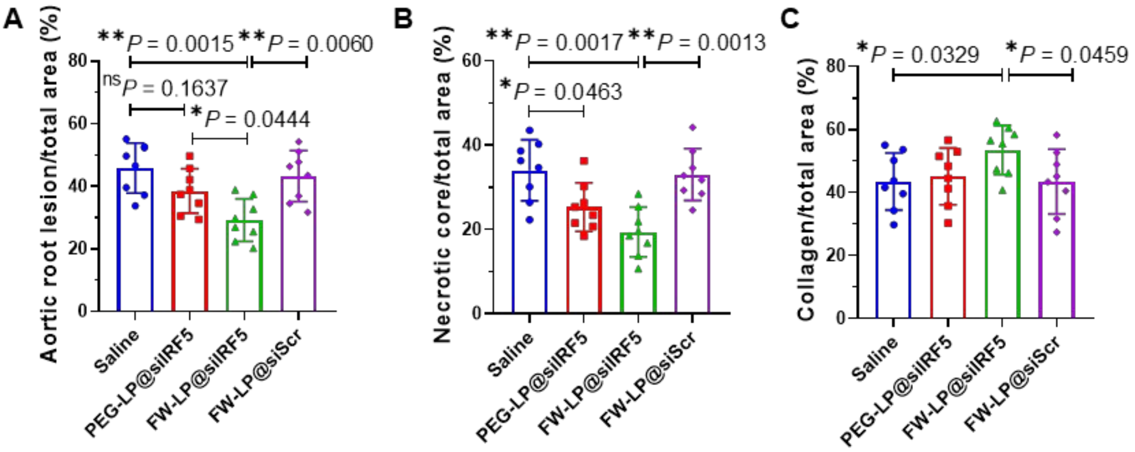

Figure S13. FW-LP@siIRF5 therapy reduces plaque burden and improves features of plaque stability. Quantitative analysis of aortic root sections from atherosclerotic mice following various treatments, including: (A) lesion area, (B) necrotic core area, and (C) collagen content, each expressed as a percentage of the total arterial wall area, measured using ImageJ software (n = 8 biologically independent mice, mean ± S.D.). Statistical significance was determined using one-way ANOVA with a Games-Howell post hoc test (A and B), or using an unpaired two-tailed t-test (C). *$P < 0.05$, **$P < 0.01$, and ns denotes no significance.

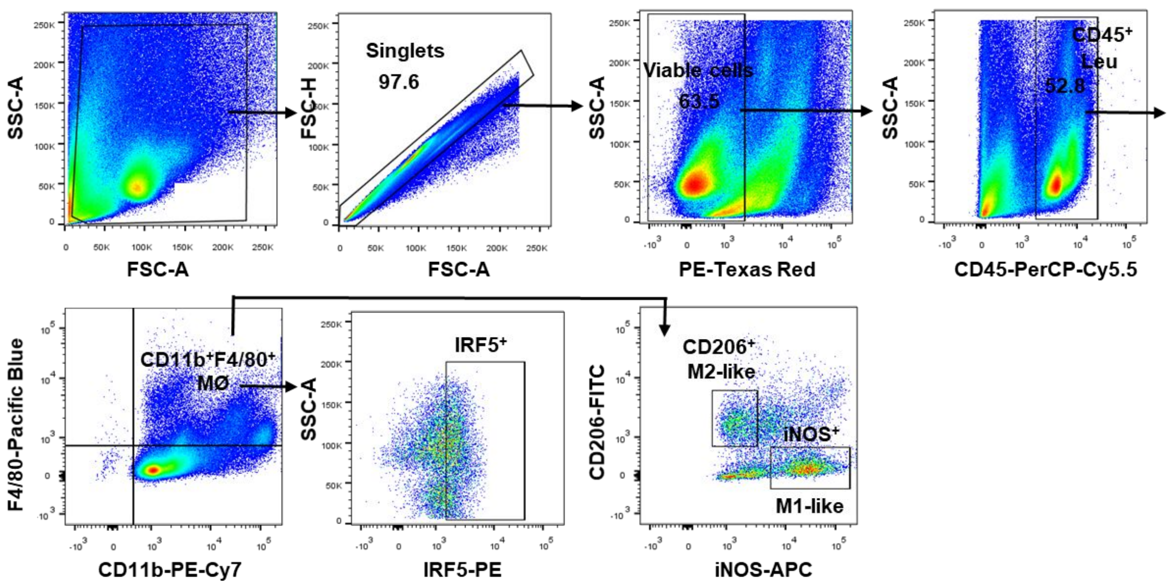

Figure S14. Flow cytometry gating strategy for IRF5 expression and lesional macrophage phenotype. Gating strategy used for flow cytometry analysis of IRF5 expression and M2-like ($CD206^+$) and M1-like ($iNOS^+$) macrophage expression levels in lesional macrophages ($CD45^+CD11b^+F4/80^+$) from digested aortas of atherosclerotic mice treated with different formulations. The IRF5 of lesional macrophages in digested aortas were identified as $CD45^+CD11b^{hi}F4/80^{hi}IRF5^+$ cells; M2-like macrophages were identified as $CD45^+CD11b^{hi}F4/80^{hi}CD206^+$ cells; M1-like macrophages were identified as $CD45^+CD11b^{hi}F4/80^{hi}$ $iNOS^+$ cells.

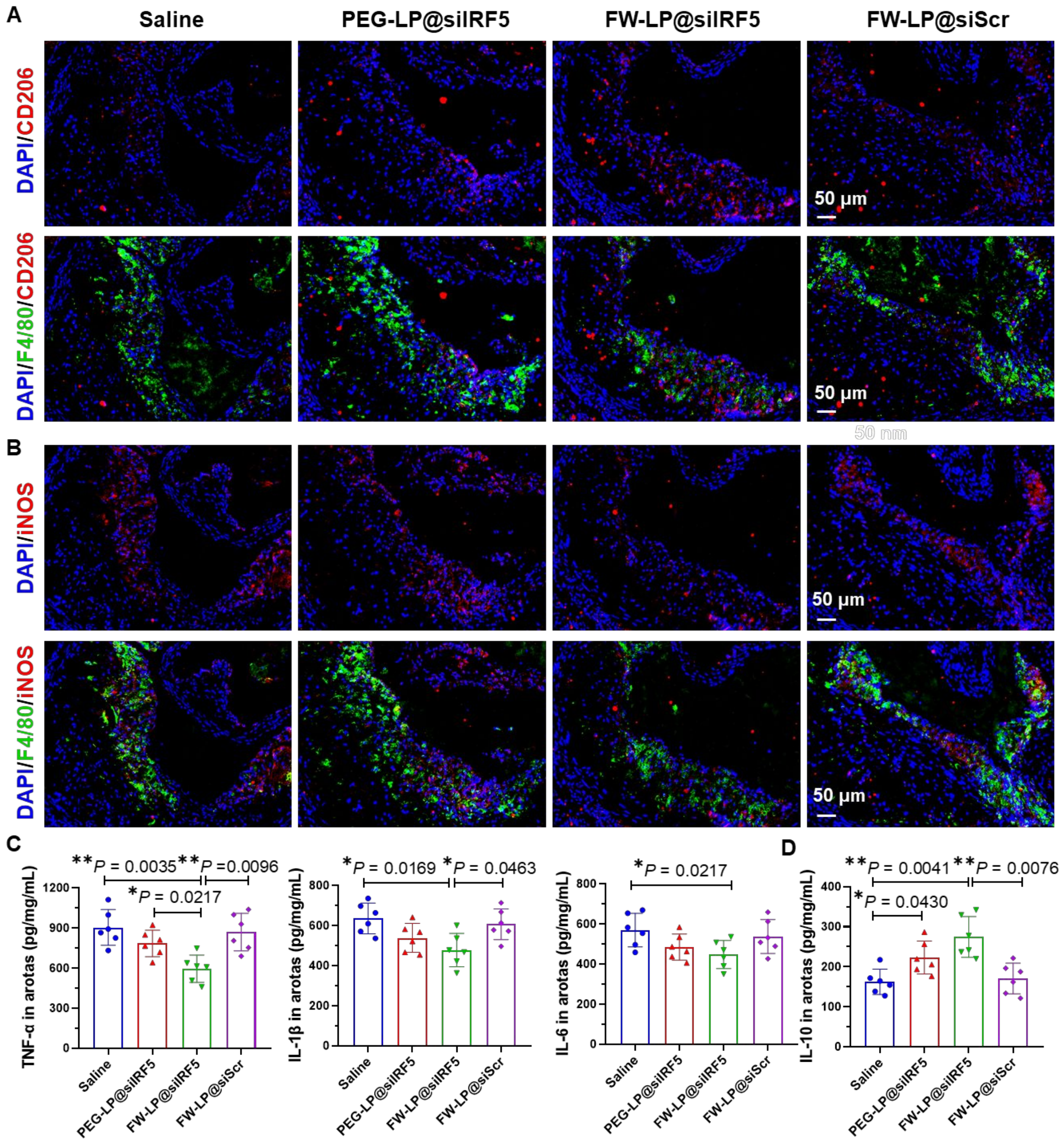

Figure S15. FW-LP@siIRF5 therapy promotes anti-inflammatory macrophage polarization and reduces inflammatory signaling. Immunofluorescence staining and cytokine profiling were used to evaluate macrophage polarization and inflammation in atherosclerotic mice treated with different formulations. (A) Representative images of F4/80$^+$CD206$^+$ macrophages (M2-like) and (B) F4/80$^+$iNOS$^+$ macrophages (M1-like) in cross-sections of aortic roots. Scale bars, 50 μm. The merge images in panels (A) and (B) are also shown in Figure 6F. (C) Levels of pro-inflammatory cytokines (TNF-α, IL-1β, and IL-6) and (D) anti-inflammatory cytokine IL-10 in aortic tissue, quantified by ELISA, after different treatments. The data were presented as mean ± S.D. (n = 6 biologically independent mice). Statistical analyses were performed using one-way ANOVA with a Games–Howell post hoc test for TNF-α, IL-1β, and IL-10, or an unpaired two-tailed t-test for IL-6. . *P < 0.05, and **P < 0.01.

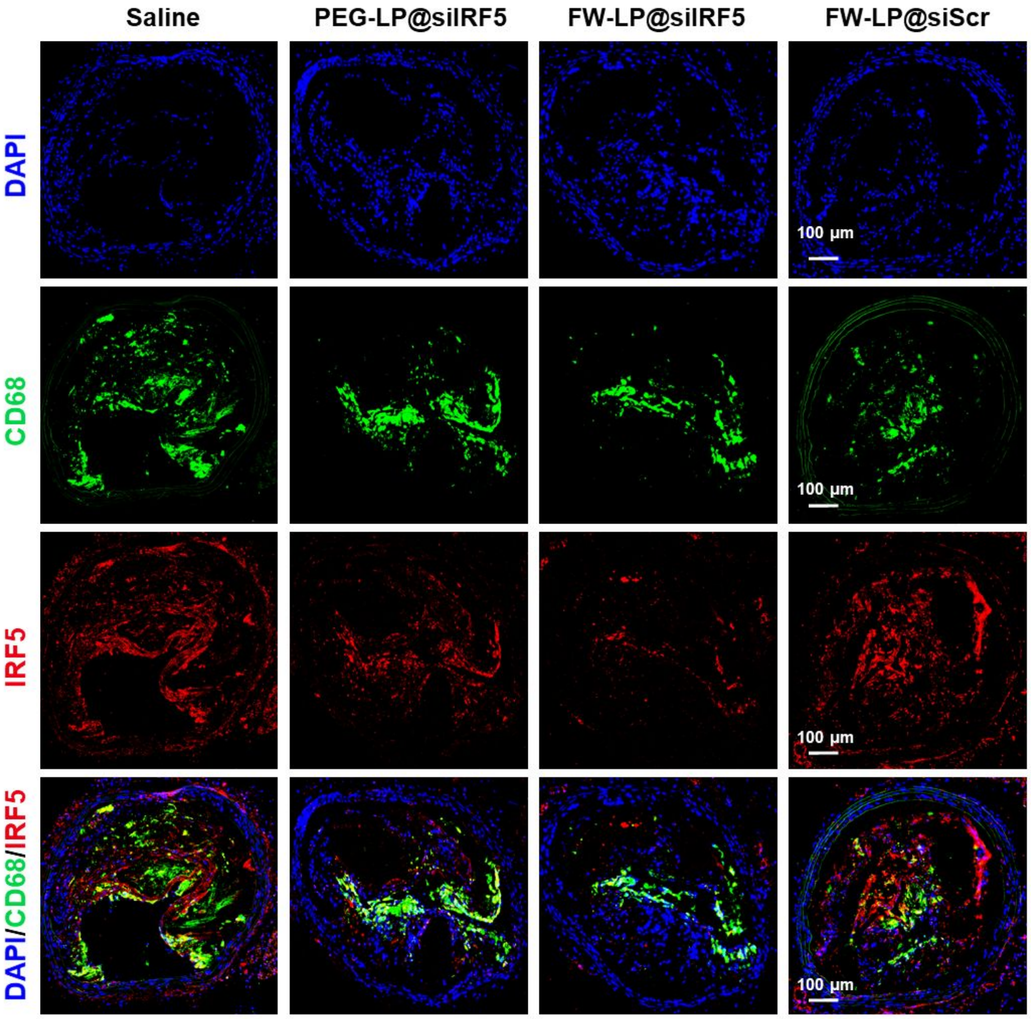

Figure S16. FW-LP@siIRF5 therapy reduces IRF5 expression in lesional macrophages of plaque-bearing, angiotensin-infused *ApoE*$^{-/-}$ mice. Twelve-week-old *ApoE*$^{-/-}$ mice were fed a HFD for 4 weeks and then received 4 additional weeks of treatment with angiotensin II infusion while continuing on the HFD. Treatments include saline or three differerent siRNA formulations (20 μg of siIRF5 or siScr per mouse). Immunofluorescence images of brachiocephalic artery sections depicting IRF5^{+} cells (IRF5, red), macrophages (CD68, green), and nuclei (DPAI, blue) within atherosclerotic plaques. The corresponding merged images are also shown in Figure 7B. Scale bars, 100 μm.

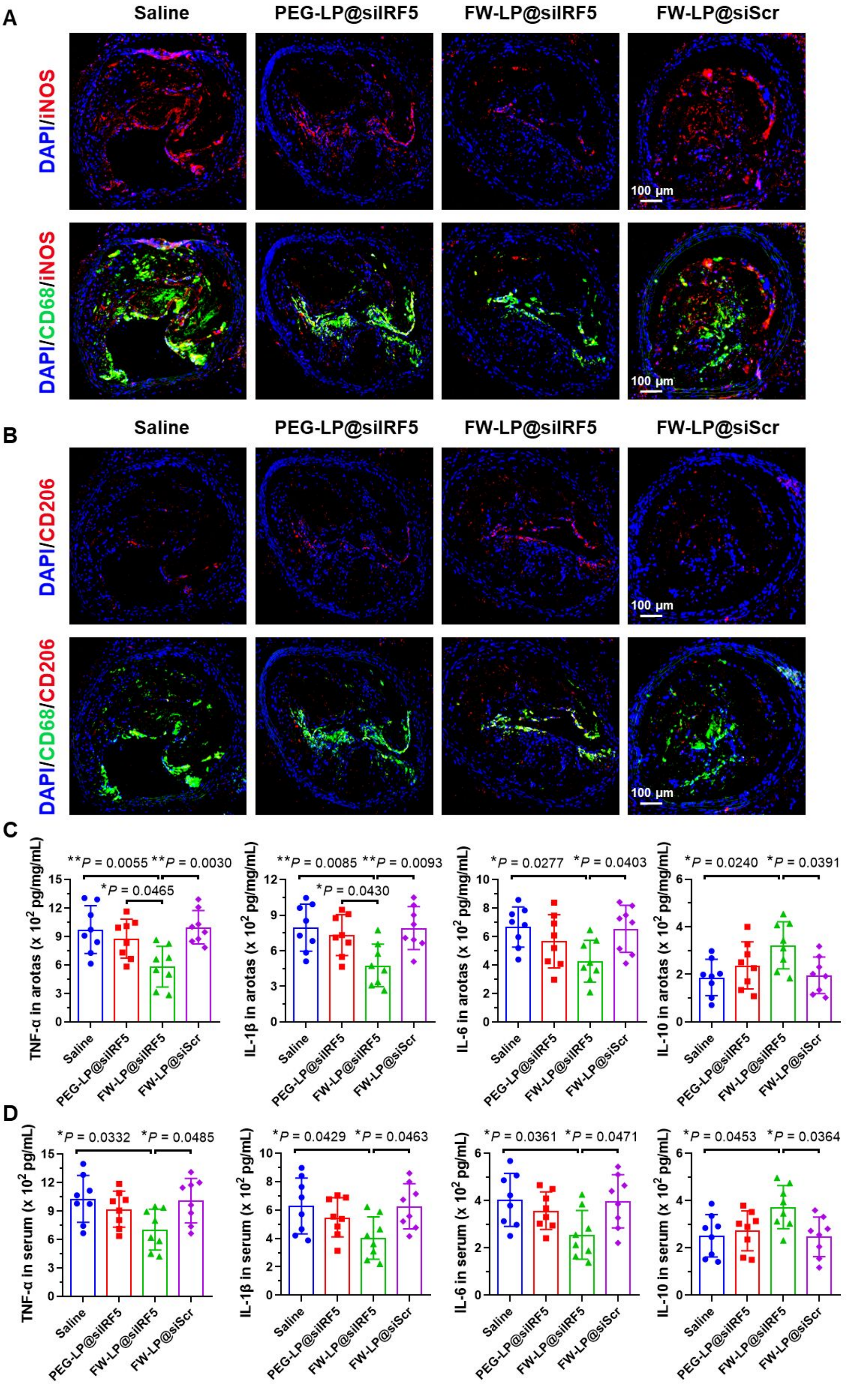

Figure S17. FW-LP@siIRF5 therapy promotes anti-inflammatory macrophage polarization and reduces inflammatory signaling in plaque-bearing, angiotensin-infused *ApoE$^{-/-}$* mice. Immunofluorescence staining and cytokine profiling were used to evaluate

macrophage polarization and inflammation following treatment with different formulations. (A) Immunofluorescence images of $CD68^+iNOS^+$ macrophages (M1-like) and (B) $CD68^+CD206^+$ macrophages (M2-like) in in brachiocephalic artery plaque sections. Scale bars, 100 µm. The corresponding merged images for panels (A) and (B) are shown in Figure 7I. (C) Aortic tissue and (D) serum levels of pro-inflammatory cytokines (TNF-α, IL-1β, and IL-6) and the anti-inflammatory cytokine IL-10 were quantified in plaque-bearing, angiotensin-infused atherosclerotic mice after various treatments (n = 8 biologically independent mice). Data were analyzed using one-way ANOVA with a Tukey post hoc test, and presented as mean ± S.D. $*P < 0.05$, and $**P < 0.01$.

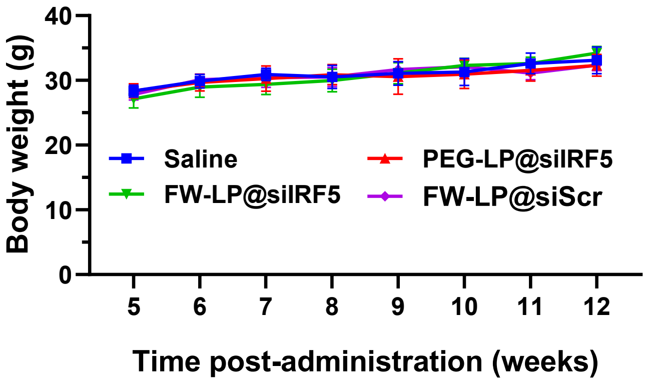

Figure S18. Time-course body weight of atherosclerotic *ApoE*$^{-/-}$ mice during various treatments. Eight-week HFD-fed *ApoE*$^{-/-}$ were injected intravenously with saline or differerent siRNA nanomedicines (PEG-LP@siIRF5, FW-LP@siIRF5, and FW- LP@siScr; siRNA, 20 µg per mouse) twice a week for an additional 8 weeks, while being maintained on the HFD. Body weight was measured weekly during the treatment period. Data were shown as mean ± S.D. (n = 8 biologically independent mice).

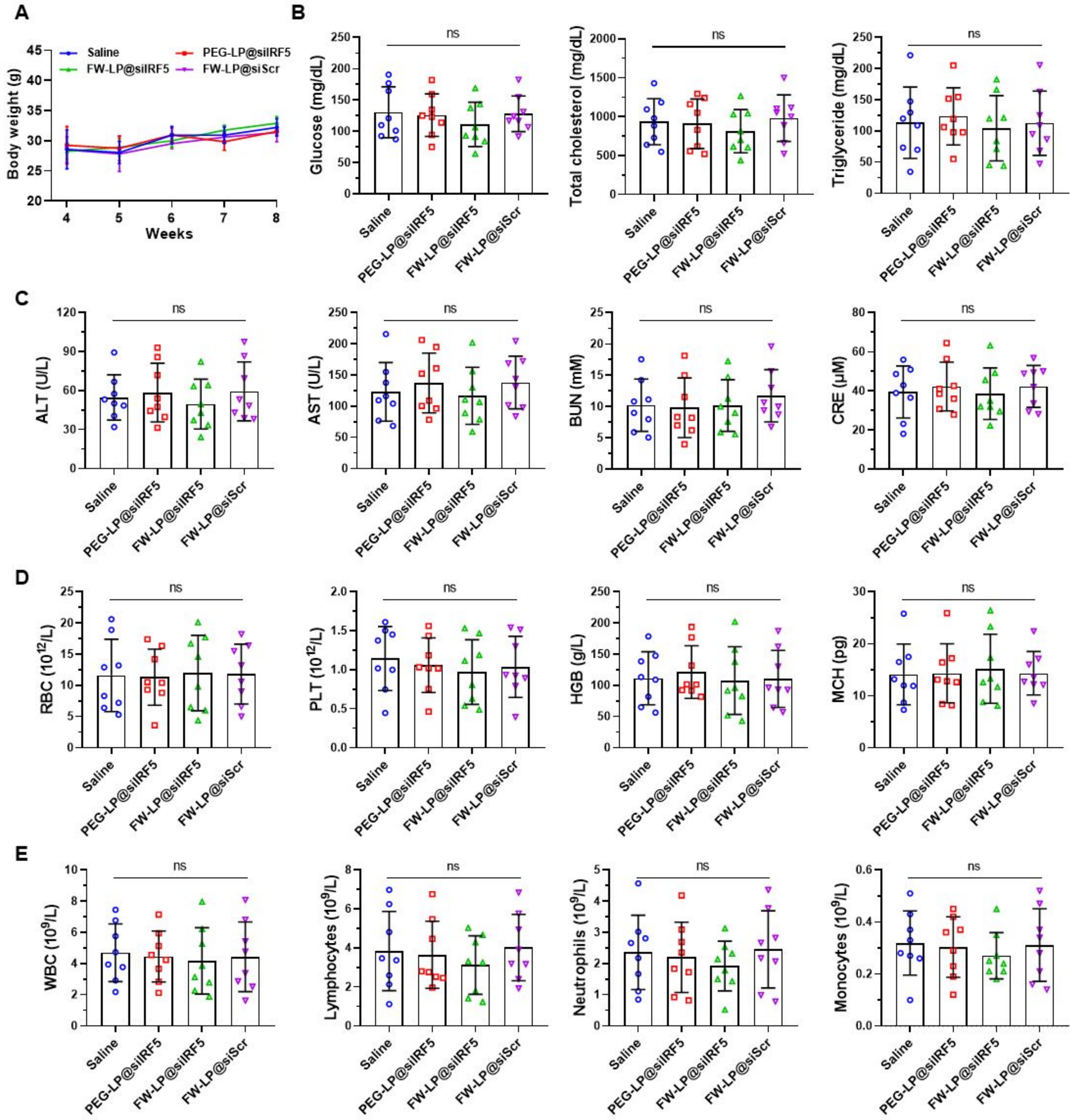

Figure S19. Biosafety Assessment in angiotensin infusion-induced atherosclerotic mice following various treatments. (A) Body weight of mice with angiotensin infusion-induced atherosclerosis over 4 weeks under various treatments. (saline and three differerent siRNA formulations; 20 μg of siIRF5 or siScr per mouse). Stable body weight indicates that FW-LP@siIRF5 treatment is well tolerated *in vivo*. (B) Serum concentrations of total cholesterol, triglyceride, and fasting blood glucose remained unchanged after FW-LP@siIRF5 treatment. There were no significant differences in the levels of serum total cholesterol, triglyceride, and fasting blood glucose, indicating that FW-LP@siIRF5 treatment did not alter the levels of cholesterol and blood glucose *in vivo*. (C) Serum liver function biomarkers (ALT and AST) and kidney function biomarkers (BUN and CRE) were assessed in *ApoE*$^{-/-}$ mice with angiotensin II infusion-induced atherosclerosis after 4 weeks of treatments. FW-LP@siIRF5 treatment did not induce hepatorenal toxicity *in vivo*. (D-E) Hematological parameters were

further evaluated including (D) red blood cell count (RBC), platelet (PLT), hemoglobin (HGB), mean corpuscular hemoglobin (MCH), and (E) immune-relatedcell counts (leukocyte, lymphocyte, neutrophil, and monocyte). FW-LP@siIRF5 treatment did not adversely affect circulatory or immune function. All data are presented as mean ± S.D. of 8 biologically independent animals. Statistical significance was performed using one-way ANOVA with a Tukey's post hoc test (n.s., not significant).

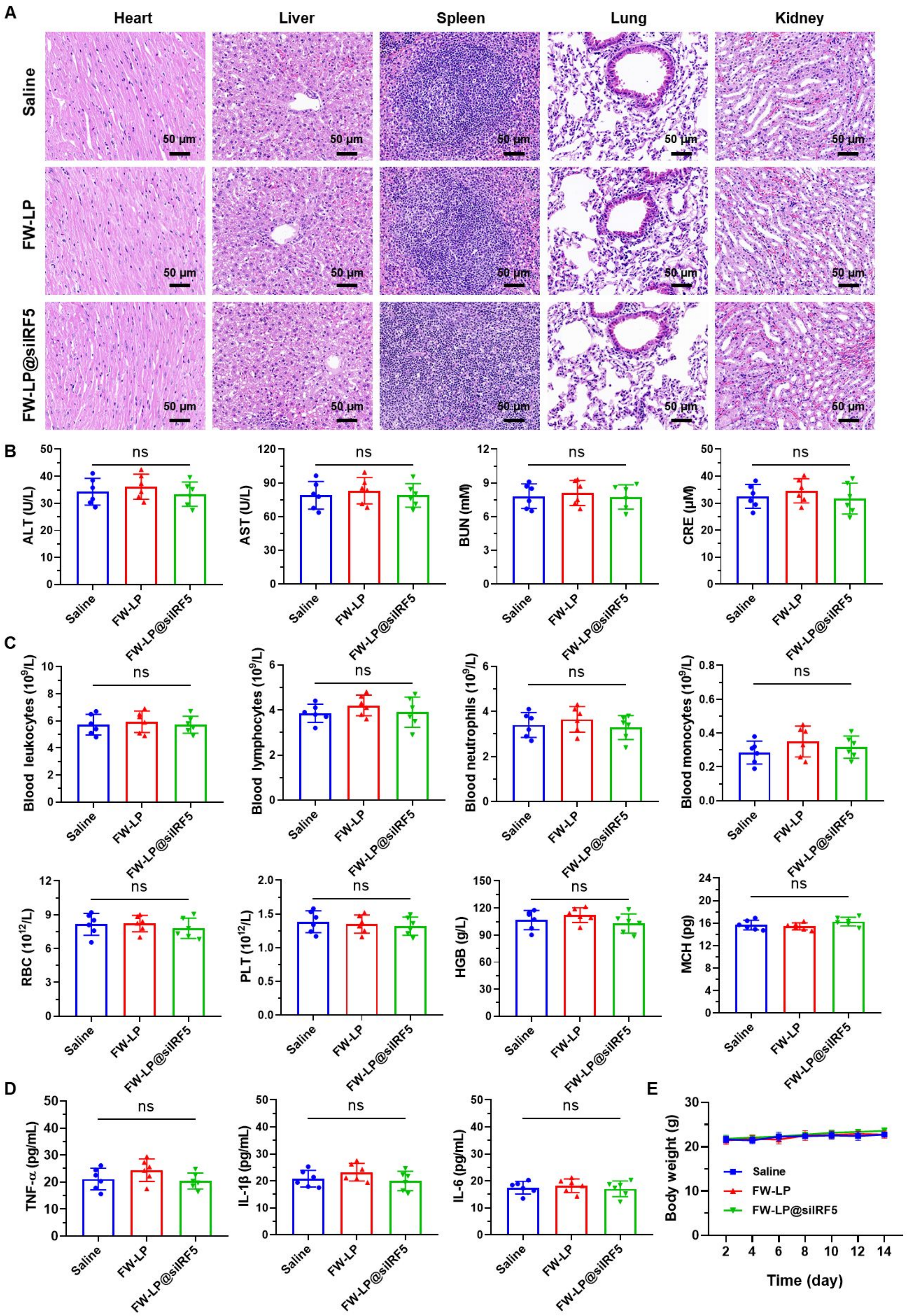

Figure S20. *In vivo* evaluation of adverse immune effects and biocompatibility of empty FW-LP or FW-LP@siIRF5. Seven-week-old C57/BL6J mice were injected intravenously

with saline, empty FW-LP, or FW-LP@siIRF5 every other day for 14 days. Forty-eight hours after the final treatment, mice were sacrificed, and blood and major organs (heart, liver, spleen, lung, and kidney) were harvested for comprehensive biosafety and immune evaluation. (A) H&E staining images of major organ sections, including heart, liver, spleen, lung, and kidney, form the C57BL/6J mice after various treatments for 14 days. Scale bars, 50 μm. (B) Serum biochemical assays evaluating liver and kidney functions, including alanine aminotransferase (ALT), aspartate aminotransferase (AST), blood urea nitrogen (BUN), and creatinine (CRE) levels (n = 6 biologically independent mice). (C) Hematological parameters including leukocytes, lymphocytes, neutrophils, monocytes, red blood cell count (RBC), platelet (PLT), hemoglobin concentration (HGB), and mean corpuscular hemoglobin (MCH) (n = 6 biologically independent mic). (D) Serum concentrations of TNF-α, IL-1β, and IL-6 after various treatments (n = 6 biologically independent mice). (E) Time-course analysis of body weight changes in treated mice during the 14-day treatment period. The data were presented as mean ± S.D. (n = 6 biologically independent mice). Statistical analyses were performed using one-way ANOVA with Tukey post hoc test. (n.s., not significant).

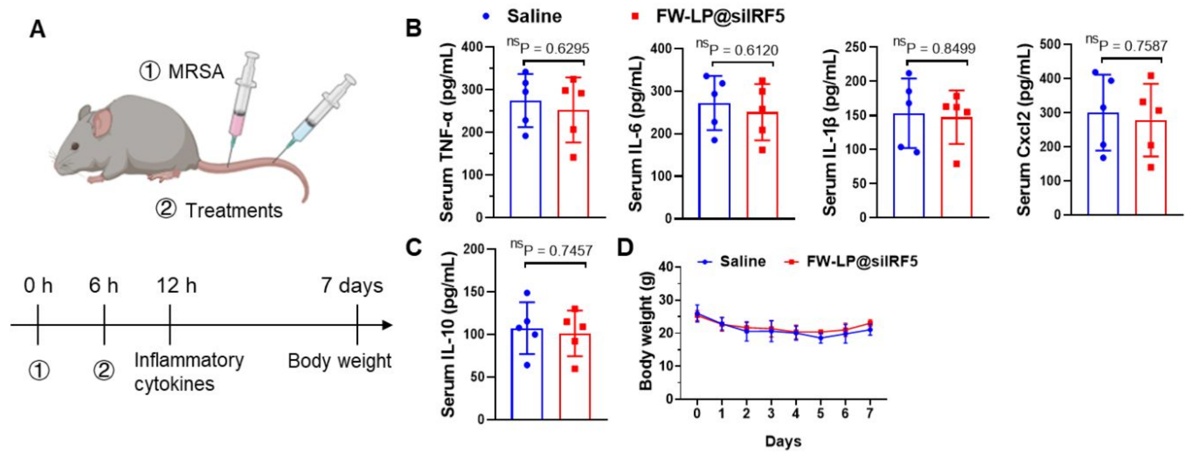

Figure S21. FW-LP@siIRF5 does not impair host inflammatory responses in an acute MRSA bacteremia model. (A) Schematic of the acute infection model. Male C57BL/6J mice (8 weeks old) were intravenously injected with MRSA (ATCC 33591; 5 × 10^7 CFU in 0.1 mL). 6 h after infection, mice were randomized to receive either saline or FW-LP@siIRF5 (50 μg per mouse; 150 μL, *i.v.*). After an additional 6 h, serum was collected for quantification of pro-inflammatory cytokines and chemokine (TNF-α, IL-6, IL-1β, and Cxcl2) and the anti-inflammatory cytokine IL-10. Body weight and survival were monitored for 7 days. (B) Serum levels of pro-inflammatory cytokines and chemokine and (C) IL-10 in MRSA-infected mice after treatment. No significant differences were detected between saline- and FW-LP@siIRF5-treated groups. (D) Time-dependent mouse body weight over 7 days in the acute MRSA bacteremia model. Data are presented as mean ± S.D. (n = 5 biologically independent animals). Statistical significance was assessed using an unpaired two-tailed Student's t-test (B and C); P > 0.05 indicates not significant.

3. Supplemental Tables

Table S1. Characteristics of LPs and LPs@siRNA nanoparticles measured by DLS.

PEG-LP referes to unloaded (siRNA-free) cationic liposome and uncoated FA-PEG$_{2k}$-Chol and WRK-PEG$_{2k}$-Chol**;** FW-LP denotes the unloaded (siRNA-free) cationic liposome-coated with FA-PEG$_{2k}$-Chol and WRK-PEG$_{2k}$-Chol; PEG-LP@siIRF5 and FW-LP@siIRF5 represent PEG-LP and FW-LP liposomes, respectively, complexed with siIRF5, respectively. Average hydrodynamic diameter, polydispersity index (PDI), and zeta potential of each formulation were determined by dynamic light scattering (DLS).

	Average Hydrodynamic Diameter by DLS (nm)	Polydispersity Index (PDI)	Zeta Potential (mV)
PEG-LP	70.1 ± 4.0	0.19 ± 0.02	13.2 ± 4.1
FW-LP	80.7 ± 8.3	0.11 ± 0.05	15.1 ± 5.4
PEG-LP@siIRF5	102.7 ± 10.8	0.28 ± 0.03	4.2 ± 1.1
FW-LP@siIRF5	120.4 ± 6.2	0.21 ± 0.04	5.4± 1.4

Table S2. Primer sequences used for the RT-qPCR analysis.

Gene	Forward primer (5′-3′)	Reverse primer (5′-3′)
PPAR-γ	AAGCCGTGCAAGAGATCACA	TGGTCATGAATCCTTGGCCC
STAT1	GCCTCTCATTGTCACCGAAGAAC	TGGCTGACGTTGGAGATCACCA
STAT6	CTCTGTGGGGCCTAATTTCCA	CATCTGAACCGACCAGGAACT
NF-κB	GACACGACAGAATCCTCAGCATCC	CCACCAGCAGCAGCAGACATG
GAPDH	TAGAGGGACAAGTGGCGTTC	CGCTGAGCCAGTCAAGTGT

Table S3. Antibodies used for the flow cytometry analysis of lesional macrophages.

Antibody	Cat#	Concentration for use
PerCP/Cyanine5.5 anti-mouse CD45	BD Biosciences; 550994	3.0 μg/mL
PE/Cyanine7 anti-mouse CD11b	BD Biosciences; 552850	2.0 μg/mL
BV421 anti-mouse F4/80	BD Biosciences; 565411	2.5 μg/mL

Table S4. Antibodies used for the flow cytometry analysis of blood cells.

Antibody	Cat#	Concentration for use
PerCP/Cyanine5.5 anti-mouse CD45	BD Biosciences; 550994	3.0 μg/mL
FITC anti-mouse CD90.2	BD Biosciences; 561973	1.5 μg/mL
FITC anti-mouse CD45R	BD Biosciences; 553088	2.5 μg/mL
FITC anti-mouse CD49b	BD Biosciences; 553857	2.5 μg/mL
FITC anti-mouse NK1.1	BD Biosciences; 553164	2.5 μg/mL
FITC anti-mouseTer119	BioLegend; 116206	2.5 μg/mL
FITC anti-mouse Ly6G	BioLegend; 127606	2.5 μg/mL
PE/Cyanine7 anti-mouse CD11b	BD Biosciences; 552850	3.0 μg/mL
BV605 anti-mouse Ly-6C	BD Biosciences; 563011	2.5 μg/mL

Table S5. Antibodies used for the flow cytometry analysis of IRF5 expression and M2-like and M1-like macrophages expression in aortic lesional macrophages.

Antibody	Cat#	Concentration for use
PerCP/Cyanine5.5 anti-mouse CD45	BD Biosciences; 550994	2.0 μg/mL
PE/Cyanine7 anti-mouse CD11b	BD Biosciences; 552850	2.0 μg/mL
BV421 anti-mouse F4/80	BD Biosciences; 565411	2.5 μg/mL
PE anti-mouse IRF5	BioLegend; 158604	2.5 μg/mL
FITC anti-mouse CD206 (MMR)	BioLegend; 141704	2.5 μg/mL
Alexa Fluor® 647 anti-mouse iNOS	CST; #48866	1.5 μg/mL

Table S6. Composition of LPs formulations.

Formulations	DOTAP (mol%)	Chol (mol%)	mPEG$_{2k}$-Chol (mol%)	FA-PEG$_{2k}$-Chol (mol%)	WRK-PEG$_{2k}$-Chol (mol%)
FW-LP	50	45	2	1	2
PEG-LP	50	45	5	0	0
FA-LP	50	45	4	1	0
WRK-LP	50	45	3	0	2

Table S7. Primer sequences used for the RT-qPCR analysis

Gene	Forward primer (5′-3′)	Reverse primer (5′-3′)
IRF5	AATACCCCACCACCTTTTG	TTGAGATCCGGGTTTGAGAT
GAPDH	TAGAGGGACAAGTGGCGTTC	CGCTGAGCCAGTCAAGTGT
